# Population structure of *Escherichia coli* O26 : H11 with recent and repeated *stx2* acquisition in multiple lineages

**DOI:** 10.1099/mgen.0.000141

**Published:** 2017-11-21

**Authors:** Yoshitoshi Ogura, Yasuhiro Gotoh, Takehiko Itoh, Mitsuhiko P. Sato, Kazuko Seto, Shyuji Yoshino, Junko Isobe, Yoshiki Etoh, Mariko Kurogi, Keiko Kimata, Eriko Maeda, Denis Piérard, Masahiro Kusumoto, Masato Akiba, Kiyoshi Tominaga, Yumi Kirino, Yuki Kato, Katsuhiko Shirahige, Tadasuke Ooka, Nozomi Ishijima, Ken-ichi Lee, Sunao Iyoda, Jacques Georges Mainil, Tetsuya Hayashi

**Affiliations:** ^1^​ Kyushu University, Fukuoka, Japan; ^2^​ Tokyo Institute of Technology, Tokyo, Japan; ^3^​ Osaka Prefectural Institute of Public Health, Osaka, Japan; ^4^​ Miyazaki Prefectural Institute for Public Health and Environment, Miyazaki, Japan; ^5^​ Toyama Institute of Health, Toyama, Japan; ^6^​ Fukuoka Institute of Health and Environmental Sciences, Fukuoka, Japan; ^7^​ Universitair Ziekenhuis Brussel, Brussels, Belgium; ^8^​ National Institute of Animal Health, National Agriculture and Food Research Organization, Ibaraki, Japan; ^9^​ National Institute of Animal Health, Ibaraki, Japan; ^10^​ Yamaguchi Prefectural Institute of Public Health and Environment, Yamaguchi, Japan; ^11^​ University of Miyazaki, Miyazaki, Japan; ^12^​ University of Tokyo, Tokyo, Japan; ^13^​ Kagoshima University, Kagoshima, Japan; ^14^​ National Institute of Infectious Diseases, Tokyo, Japan; ^15^​ University of Liège, Liège, Belgium

**Keywords:** enterohaemorrhagic *Escherichia coli*, Shiga toxin, genome evolution, population structure, antimicrobial-resistance gene

## Abstract

A key virulence factor of enterohaemorrhagic *Escherichia coli* (EHEC) is the bacteriophage-encoded Shiga toxin (Stx). Stxs are classified into two types, Stx1 and Stx2, and Stx2-producing strains are thought to cause more severe infections than strains producing only Stx1. Although O26 : H11 is the second most prevalent EHEC following O157 : H7, the majority of O26 : H11 strains produce Stx1 alone. However, Stx2-producing O26 strains have increasingly been detected worldwide. Through a large-scale genome analysis, we present a global phylogenetic overview and evolutionary timescale for *E. coli* O26 : H11. The origin of O26 has been estimated to be 415 years ago. Sequence type 21C1 (ST21C1), one of the two sublineages of ST21, the most predominant O26 : H11 lineage worldwide, emerged 213 years ago from one of the three ST29 sublineages (ST29C2). The other ST21 lineage (ST21C2) emerged 95 years ago from ST21C1. Increases in population size occurred in the late 20th century for all of the O26 lineages, but most remarkably for ST21C2. Analysis of the distribution of *stx2*-positive strains revealed the recent and repeated acquisition of the *stx2* gene in multiple lineages of O26, both in ST21 and ST29. Other major EHEC virulence genes, such as type III secretion system effector genes and plasmid-encoded virulence genes, were well conserved in ST21 compared to ST29. In addition, more antimicrobial-resistance genes have accumulated in the ST21C1 lineage. Although current attention is focused on several highly virulent ST29 clones that have acquired the *stx2* gene, there is also a considerable risk that the ST21 lineage could yield highly virulent clones.

## Abbreviations

AMR, antimicrobial resistance; EHEC, enterohaemorrhagic Escherichia coli; HPD, highest posterior density; HUS, haemolytic-uraemic syndrome; LEE, locus of enterocyte effacement; MGE, mobile genomic element; ML, maximum likelihood; SNP, single nucleotide polymorphism; ST, sequence type; Stx, Shiga toxin; T3SS, type III secretion system; TMRCA, time to themost recent common ancestor; VF, viruence factor; WG, whole genome.

## Data Summary

The raw read sequences and assembled scaffold sequences obtained in this study have been deposited in GenBank/EMBL/DDBJ under the BioProject accession number PRJDB5579. Six supplementary tables and four supplementary figures are available with the online Supplementary Material.

## Impact Statement

*Escherichia coli* O26 : H11 is the second most prevalent enterohaemorrhagic *E. coli* (EHEC). Production of Shiga toxin 2 (Stx2), which is one of the two subtypes of Stx, is thought to be a risk factor for severe EHEC infection, and the emergence and spread of several O26 : H11 clones that produce Stx2 are of great concern globally. These clones belong to sequence type 29 (ST29). Here, we present a global timescaled phylogenomic view of O26 : H11, with evidence of the recent and repeated acquisition of *stx2* by multiple ST29 lineages, as well as the O26 : H11 ST21 lineage. Other EHEC virulence-related genes are well conserved and more antimicrobial-resistance genes have accumulated in ST21. These findings indicate that this lineage also requires intensive surveillance for the emergence of *stx2*-positive, highly virulent clones.

## Introduction

Enterohaemorrhagic *Escherichia coli* (EHEC) is a major cause of serious gastrointestinal illness, which includes diarrhoea, haemorrhagic colitis and life-threatening haemolytic-uraemic syndrome (HUS) [1]. Ruminant animals, bovines in particular, are thought to serve as the main reservoirs of EHEC, with human infections likely occurring through the consumption of contaminated foods, such as meats and dairy products, as well as direct animal-to-human and human-to-human transmissions [2]. Typical EHEC strains produce Shiga toxins (Stxs) encoded by lysogenic bacteriophages integrated as prophages, possess a pathogenicity island called the locus of enterocyte effacement (LEE), and a large virulence plasmid encoding enterohaemolysin and other potential virulence factors (VFs) [1]. Stxs are divided into two major groups, Stx1 and Stx2, and both are further classified into several subtypes. EHEC strains that cause human diseases typically harbour one or more subtypes, including *stx1a*, *stx2a*, *stx2c* and *stx2d*; *stx2a*-positive strains are more often associated with severe disease [3, 4]. The LEE encodes a set of proteins constituting a type III secretion system (T3SS), as well as several effectors secreted by the T3SS. More than 30 T3SS effectors have also been found in non-LEE genomic regions, mostly on prophage genomes (non-LEE effectors) [5, 6].

Although O157:H7 is the most predominant serotype, EHEC strains of many other serogroups cause outbreaks and sporadic cases worldwide [1–3]. The four major EHEC serogroups (O157, O26, O111 and O103) are phylogenetically divergent, but share a similar VF set [6]. These EHECs all harbour a large number of mobile genomic elements (MGEs), including many prophages and a virulence plasmid. MGEs carrying the same VF genes were found in each EHEC, but many of them showed distinct evolutionary histories, indicating that the independent acquisition of these MGEs has driven the parallel evolution of EHEC pathogenesis [6–8].

Among non-O157 EHECs, O26 : H11 strains are those most frequently associated with human diseases. They have been increasingly detected in Japan, the USA, Australia and many European countries [9–12]. The European Food Safety Authority (EFSA) and the European Centre for Disease Prevention and Control (ECDC) reported that in food samples, the detection of O26 : H11 has increased recently, and in 2015 the proportion of O26 : H11 was almost equal to that of O157:H7 [10]. Strains of O26 : H11 can cause symptoms as severe as those caused by O157 : H7 [13, 14]. Although the majority of O26 : H11 strains isolated from patients harbour *stx1a* only [11, 12, 15, 16], isolates of strains containing *stx2a* have been increasingly reported in many countries [12, 17–20]. It has been hypothesized that *stx2a*-positive strains have a higher potential to cause HUS compared to strains carrying *stx1a* only [21]. In fact, a highly virulent *stx2*-positive O26 : H11 clone was recently identified as an emerging cause of HUS in Europe (referred to as the ‘new European clone’), and it has disseminated around the world [18, 21–23].

Multi-locus sequence typing showed that most O26 : H11 strains were divided into two closely related sequence types (STs), ST21 and ST29. While the most predominant ST among O26 : H11 clinical isolates is ST21 [18, 24–26], and most *stx1a*-positive strains belong to ST21, the new European clone belongs to ST29 [21]. The presence/absence profiles of four plasmid-encoded VF (pVF) genes, *ehxA* (enterohaemolysin), *katP* (catalase peroxidase), *espP* (serine protease) and *etpD* (effector of type II secretion system), can be used to distinguish ST21 and ST29, and further classify both STs into several clonal lineages [18, 21]. This profiling system has been used in several studies [26–28]. The new European clone exhibits the following pVF gene profile: *ehxA+/katP−/espP−/etpD+* [21]. More recently, two additional virulent ST29 clones were identified, and each clone exhibited a distinct profile: *ehxA−/katP−/espP−/etpD−* or *ehxA+/katP−/espP+/etpD−*. The former clone was originally identified as *stx2d*-positive strains that were isolated from paediatric patients in France (named the ‘new French clone’) [27]. Strains belonging to this clonal lineage with no *stx* genes were also isolated from bovine faeces in the USA [29, 30]. The latter clone harbours *stx2a* alone and has been shown to produce a higher concentration of Stx2 toxin and to exhibit a higher virulence in mice compared to strains belonging to the new European clone and ST21 [18]. Importantly, however, Bielaszewska *et al*. reported that *stx2a*-positive ST21 strains do not substantially differ in their association with HUS from *stx2a*-harbouring ST29 strains, and they therefore concluded that the possession of *stx2a* rather than the ST of the strain is a predictor for HUS development in O26 infection [21].

To date, the clonal diversity of O26 : H11 has been analysed using several different typing systems, such as multi-locus sequence typing, multiple-locus variable number tandem repeat analysis, multiplex single nucleotide polymorphism (SNP) analysis, pulsed-field gel electrophoresis, clustered regularly interspaced short palindromic repeat (CRISPR) typing and plasmid VF gene profiling [21, 22, 27, 28, 30–35]. However, these typing systems do not have enough resolution power to discriminate closely related strains and/or do not reveal the precise phylogenetic relationships between strains. Although whole genome (WG)-based high-resolution phylogenetic analyses of O26 : H11 strains have been conducted in a few studies, the numbers of strains analysed were rather limited (37 strains by Ishijima *et al.* [18] and 79 by Gonzalez-Escalona *et al*. [29]). To present a phylogenetic overview of O26 : H11, we performed a WG-based phylogenetic analysis of more than 500 strains isolated in eight countries, including bovine isolates and strains with the enteropathogenic *E. coli* (EPEC) pathotype (negative for *stx*). We subsequently conducted temporal and population structural analyses of each clonal lineage and investigated the prevalence of genes for Stxs, known T3SS effectors, pVFs and antimicrobial resistance (AMR) in the O26 strains.

## Methods

### Bacterial strains and DNA sequencing

The 520 O26 strains used in this study are summarized in Table 1. The strains sequenced in this study were mainly isolated in Japan from humans (*n*=252) and bovines (*n*=32) from 1994 to 2013. We also sequenced human and bovine isolates from Belgium (*n*=30), the USA (*n*=19), France (*n*=3), The Netherlands (*n*=1), Switzerland (*n*=1), the UK (*n*=1) and Italy (*n*=1). From the public database, the genome information of O26 strains isolated from humans and bovines in the USA (*n*=72), the UK (*n*=57), Japan (*n*=29), The Netherlands (*n*=14) and France (*n*=8) were included. Details of the 340 strains sequenced in this study and of the 180 strains with publicly available genome data are given in Tables S1 and S2 (available in the online Supplementary Material), respectively.

**Table 1.** O26 strains used in this study

**Country**	**Host**	**Year of isolation**	**No. of strains**	**Sequence data source**
Japan	Human	1994–2011	252	This study
Japan	Bovine*	2001–2013	32	This study
Belgium, USA, France, Switzerland, UK	Human	1952–2013	29	This study
Belgium, USA, Italy, The Netherlands	Bovine	1987–2012	27	This study
Japan, USA, France, The Netherlands, UK	Human	1997–2016	137	Public database
USA	Bovine†	1983–2011	43	Public database
Total			520	

*One sheep isolate is included.

†One pig isolate is included.

Genomic DNA was purified from 1 ml overnight culture of each strain using a DNeasy blood and tissue kit (Qiagen). Genomic DNA libraries were prepared from each strain using the Nextera XT DNA sample preparation kit (Illumina), and sequenced using the Illumina HiSeq and MiSeq platforms to generate 100 and 300 bp paired-end reads, respectively.

### Genome assembly, SNP detection and phylogenetic analysis

Genomic assembly, scaffolding and gap-closing of the Illumina sequence reads obtained in this study and from the public database were performed using the Platanus assembler [36]. For strains with public sequence data, the original assembly was used if available for further analysis. Scaffold sequences of each strain were aligned with the phage- and IS-masked chromosome sequence of O26 strain 11 368 using the MUMmer [37] sequence alignment package to identify the conserved regions (cut-off threshold >98 % sequence identity and >1000 bp alignment length) of these strains and the SNP sites located in the conserved regions. We then combined all the alignment results and identified a 3 121 447 bp sequence of the strain 11 368 chromosome that was conserved in all of the strains examined (core genome) by using our in-house programs (all providable upon request). The genome sequences of each test strain were reconstructed using the SNP information and subjected to recombination analysis by Gubbins [38]. Gubbins detected and removed 194 recombinogenic sites. Nine strains in which the recombination-free core genome alignment was completely identical to that of at least one other strain were removed for further analyses (these strains are not listed in Tables S1 and S2). Finally, RAxML [39] was used to reconstruct a maximum-likelihood (ML) phylogenetic tree inferred from the concatenated alignment of 16 346 recombination-free SNP sites located on the core genome with the General Time Reversible (GTR)-GAMMA model of nucleotide substitution and 500 bootstraps. All ML trees were displayed and annotated using iTOL [40]. Clustering analysis was performed using the 16 346 bp sequences and the hierBAPS (the hierarchical Bayesian analysis of population structure) program [41]. Parameters used were (i) two levels in the hierarchy (L) and (ii) a maximum number of cluster (maxK) of 15.

### Temporal analysis

To perform temporal analysis of the ST21C1, ST21C2 and ST29 lineages individually, we identified recombination-free informative SNP sites on the core backbone of each lineage, as described above. In each lineage, 7018 SNP sites in ST21C1 (core genome size 3 899 261 bp), 8248 in ST21C2 (4 024 434 bp) and 5486 in ST29 (3 787 453 bp) were identified. Then, we investigated the temporal signals in ML trees for each O26 lineage using TempEst [42] to assess the linear relationship between the root-to-tip distance and the year of isolation. We performed further temporal analysis to date the important nodes using beast (version 1.8), a Bayesian phylogenetic inference software [43]. The GTR model of nucleotide substitution was chosen as a best fit model under the Akaike information criterion (AIC) using MrModeltest2 (https://github.com/nylander/MrModeltest2) implemented in paup* (http://paup.csit.fsu.edu/). We compared four combinations of different clock types (strict clock and uncorrelated relaxed clock) and population models (constant and Bayesian skyline). The isolation date in years was used to calibrate the timescale of the tree. Three independent Markov chain Monte Carlo (MCMC) analyses were run, each with a 10 million burn-in and 100 million chain length, sampled every 10 000 states using beagle [44] in conjunction with beast. The three runs of each lineage were combined with LogCombiner (implemented in beast) with the first 10 % of the stats in each chain removed as a burn-in. The maximum clade credibility tree was summarized using TreeAnnotator (implemented in beast), followed by visualization with Figtree (http://tree.bio.ed.ac.uk/software/figtree/). In all cases, a strict clock with a prior Bayesian skyline coalescent demographic proved to be the best-fit model based on the AIC through MCMC (AICM) as estimated by Tracer (http://tree.bio.ed.ac.uk/software/tracer/). To further assess the existence of temporal structure in the data, we performed a date randomization test, as described elsewhere [45, 46]. We prepared 10 date-randomized replicates for each lineage and analysed them by beast with the same parameters described above. The Bayesian skyline plots were calculated and visualized using Tracer to investigate changes in the effective population sizes of the three O26 lineages. The effective sample sizes were above 200 for all of the parameters.

### ST typing, *stx* and *eaeA* subtyping, and plasmid gene profiling

STs of each strain were determined based on the sequences of seven housekeeping genes (*adk*, *fumC*, *gyrB*, *icd*, *mdh*, *purA* and *recA*), which were obtained from each draft genome by blastn search (100 % identity and 100 % coverage) using allele sequences of the seven genes obtained from EteroBase (http://enterobase.warwick.ac.uk) as queries. The detection and subtyping of *stx1, stx2* and *eaeA* were also performed by *in silico* analyses of the genome sequences using blastn (>99 % identity and >99 % coverage). Reference sequences of each subtype of *stx* and *eaeA* have been described elsewhere [47, 48]. *In silico* pVF gene profiling was conducted by blastn (≤3 base mismatches, and <2500 bp distance between forward and reverse primers) using previously published primer sequences for *ehxA* [49], *katP* [50], *espP* [51] and *etpD* [52] as query sequences.

### Repertoire analysis of other virulence, AMR and plasmid genes

The conservation of other VF genes (T3SS effector genes and pVF genes), AMR genes and plasmid genes (pO26_1 and pO26_2) were analysed using the Short Read Sequence Typing for Bacterial Pathogens (srst2) program [53] in the 429 strains in which raw read sequence data were available. A set of respective VF gene sequences from the 11 368 reference strain was used as the VF gene database. For the T3SS effector genes (*nleD* and *ospG*) and pVF genes (*etpD* and *espP*) that are absent in strain 11368, the nucleotide sequences of ECs0850 (*nleD*), pO157_003 (*etpD*) and pO157_079 (*espP*) of O157 strain Sakai (BA000007) and ECO111_1634 (*ospG*) of O111 strain 11 128 (AP010960) were used as references. For acquired AMR genes, the database file ARGannot.r1.fasta that is distributed with srst2 was used. Genes encoded on pO26_1 and pO26_2 were used as a database of plasmid genes. For pO26_1, the transposase genes were excluded. The locus_tag numbers of the T3SS effectors and pVFs used as references are listed in Table S3.

## Results and Discussion

### O26 strains analysed in this study

A total of 340 O26 : H11 strains isolated from humans and bovines in Japan, the USA and several European countries were sequenced in this study, and 180 strains with publicly available genome sequence data were also included (Tables 1, S1 and S2). All of the strains, including strains with a non-motile phenotype, contained the *fliC* H11 allele as confirmed by blastn searches. *In silico* ST typing using the draft genome sequences revealed that 437 and 76 strains belonged to ST21 and ST29, respectively, and 7 were single locus variants of ST21 or ST29 (Tables 2 and S4). All but one strain (strain TC6168) harboured the *eaeA* gene (subtype β1), which is a genetic marker of the LEE. The *stx* genotypes of most of the ST21 strains were *stx1a* alone or *stx1a/stx2a*, and those of most of the ST29 strains were *stx2a* alone or *stx* negative (Table 2). Six strains were found to carry *stx2d*. Among the 42 USA strains that were previously sequenced and reported to be *stx* negative [29], a full length *stx2a* gene was detected in seven strains by our blastn analysis (Table S2).

**Table 2.** STs and *stx* genotypes of the 520 O26 strains

	*stx1a*	*stx2a*	*stx2d*	*stx1a+2a*	*stx1a+2d*	*stx* negative	Total
ST21	353	20	2	44	1	17	437
SLV of ST21	5	1	0	0	0	0	6
ST29	1	27	3	0	0	45	76
SLV of ST29	0	0	0	0	0	1	1
Total	359	48	5	44	1	63	520

SLV, single locus variant.

### Phylogenetic overview of the O26 : H11 strains

**Fig. 1.**
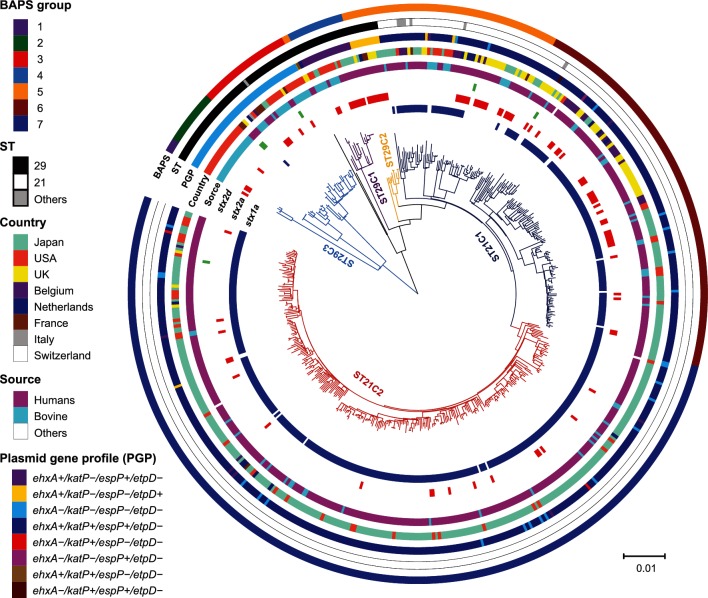
WG-based phylogenetic tree of 520 O26 strains. WG assembles of 520 O26 strains were aligned to the complete chromosome sequence of strain 11368, and the SNPs located on the 3 121 447 bp backbone sequence that were conserved in all of the test strains were identified. After removing the recombinogenic SNPs sites, the concatenated alignment of 16 346 informative sites was used to generate a ML phylogeny. From the outside in, the coloured rings represent the BAPS group; ST; plasmid gene profile (PGP); country of isolation; source of isolation; and presence of *stx2d*, *stx2a* and *stx1a*.

To obtain a phylogenetic overview of the O26 : H11 strains, we reconstructed a WG-based phylogeny of 520 O26 : H11 strains isolated worldwide based on a concatenated alignment of 16 346 recombination-free SNP sites located on the 3 121 447 bp conserved chromosomal backbone sequence of reference strain 11368 [6]. Three ST29 clonal complexes, ST29C1–ST29C3, which were previously proposed based on pVF gene profiles [18, 21], were clearly recognized in this ML tree (Fig. 1). By hierBAPS analysis [41], ST29C3 was further divided into three BAPS groups (Fig. 1). There were two ST29 strains, 248 542 and 680_13, that showed unique pVF gene profiles (*ehxA+/katP+/espP+/etpD−* and *ehxA+/katP+/espP−/etpD−*, respectively), and they grouped together with ST29C2 and several sublineages of ST21 in the hierBAPS analysis (Fig. 1).

ST21 strains separated into two large clusters named ST21C1 and ST21C2 (Fig. 1). ST21C1 was further classified into two BAPS groups, one of which was grouped together with ST29C2 and the two minor ST29 clones, as mentioned above. However, the ML tree indicated that ST21C1 emerged from a sublineage of ST29C2, and that ST21C2 appeared from a sublineage of ST21C1. Interestingly, the distribution of the ST21 strains isolated in Japan was highly biased to ST21C2 and one sublineage of ST21C1. In the Japanese-strain-enriched lineage and sublineage, only a small number of European strains (from the UK or The Netherlands) were included. This may imply that transfer of ST21 strains has not frequently occurred between Japan and European countries.

Previously, O26 : H11 strains were grouped into four different SNP clonal complexes (SNP-CC1 to SNP-CC4) by two independent studies using the same set of 48 SNPs [22, 31]. In this scheme, SNP-CC1 includes ST29C1 and ST29C3, and SNP-CC2, SNP-CC3 and SNP-CC4 correspond to ST29C2, ST21C1 and ST21C2, respectively. Based on the genetic relationships between the four SNP-CCs, Bletz *et al*. proposed that O26 : H11 evolved sequentially from SNP-CC1 to SNP-CC4 [31]. Our phylogeny supports this hypothesis (Fig. 1).

### Phylogenetic relationships of the human and bovine isolates

In O157 : H7, strains belonging to a lineage called lineage II were found to be more frequently associated with bovines than other lineages (lineages I and I/II) [54, 55]. Recently, using support vector machine analysis, Lupolova *et al*. showed that only a minor subset of bovine O157:H7 isolates were predicted to have the potential to cause human disease [56]. In O26 : H11, the bovine isolates were distributed across most of the major lineages (Fig. 1). While many bovine isolates were included in ST29C1 (9 out of 17 strains) and ST29C3 (38 out of 50 strains), no bovine isolates were included in ST29C2 (0 out of 10 strains), showing a contrasting distribution. However, the number of ST29 genomes currently available may be not sufficient to address the phylogenetic difference in the distribution of clinical and bovine isolates in ST29. In the ST21 lineage, bovine isolates were distributed rather evenly and clustered together with the clinical isolates. We detected no enrichment of bovine isolates in the specific sublineages, suggesting that, at least in ST21, the sublineages that are more frequently associated with bovines are not present.

### Temporal and population structural analyses of each O26 : H11 clade

**Fig. 2.**
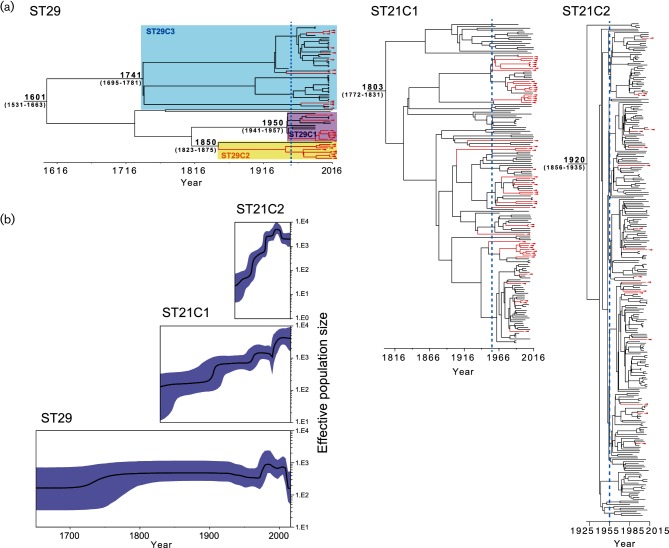
Temporal analyses of the O26 strains. (a) Results of the temporal analyses of ST29, ST21C1 and ST21C2 are shown. The time-calibrated phylogenetic trees were reconstructed using beast based on 5657, 7135 and 8391 concatenated recombination-free SNPs for ST29, ST21C1 and ST21C2, respectively. The *stx2*-carrying strains and lineages are indicated by red triangles and red lines, respectively. The dashed blue lines indicate the past 60 years (1966) in each time-stamped tree. Many Stx2 acquisition events were estimated to have occurred within the past 60 years, especially in the ST21 lineage (at least 43 % of the events in ST29, at least 90 % in ST21C1 and 100 % in ST21C2). (B) Bayesian skyline plots of ST29, ST21C1 and ST21C2 are shown. The effective population size is indicated by the black curve, and the 95 % credible interval is indicated by cyan shading.

To generate a time-stamped phylogenetic tree and to estimate the time to the most recent common ancestor (TMRCA) of each O26 : H11 clonal lineage, we performed temporal analyses of ST29, ST21C1 and ST21C2 using Bayesian coalescent analysis implemented in beast [43] (Fig. 2a). Each tree was calibrated using the isolation dates of strains, which ranged from 1952 to 2016 (ST29), from 1967 to 2016 (ST21C1) and from 1985 to 2015 (ST21C2) (Tables S1 and S2). The overall topologies of each of the generated maximum clade credibility trees were consistent with those in the ML tree (Fig. 1). Root-to-tip regression analyses revealed a weak but positive correlation between genetic distance and sampling date in each lineage (Fig. S1). In addition, date randomization tests [45, 46] supported the presence of temporal structure in our data sets (Fig. S1). In each lineage, the mean base substitution rates of randomized replicates and their 95 %credible intervals (CIs) were not within the 95 % CIs deduced with the correct sampling times.

The estimated mutation rate was 4.33×10^−7^mutations per site per year [95 % highest posterior density (HPD): 3.76–4.86×10^−7^] for ST29, 2.80×10^−7^ (2.42–3.21×10^−7^) for ST21C1 and 3.41×10^−7^ (3.01–3.86×10^−7^) for ST21C2, which were all in a range similar to those observed in other temporal analyses of *E. coli* strains (4.39×10^−7^mutations per site per year) [57]. Based on these mutation rates, the TMRCA of ST29 was estimated to be 415 years ago (95 % HPD 353–485 years). For O157 : H7, using a similar methodology, Dallman *et al.* estimated that the divergence of the contemporary β-glucuronidase-negative, sorbitol-negative clone from a β-glucuronidase-positive ancestor occurred approximately 400 years ago [58]. Thus, the MRCAs for the contemporary O26 : H11 and O157:H7 clones may have emerged in the same era. Conversely, our estimation was considerably different from that estimated by Bletz *et al*. [31]. They classified the O26 : H11 strains into four clonal complexes (SNP-CC1 to SNP-CC4) as mentioned above and postulated that EHEC O26 : H11 evolved sequentially from SNP-CC1 to SNP-CC4 within the past 1650 years. This discrepancy is probably due to the difference in methodology. The estimation by Bletz *et al*. employed a previously proposed synonymous substitution rate (1.44×10^−10^ substitutions per base per generation) [59] and generation time (300 generations per year) [60].

The estimated TMRCA of ST21C1 in the ancestral lineage of ST21C2 was 213 years ago (95 % HPD 185–244 years), and that of ST21C2 was 95 years ago (95 % HPD 80–159 years). Bayesian skyline plots showed that ST29, ST21C1 and ST21C2 each experienced several rapid increases (and a decrease) in their effective population sizes since their emergence (Fig. 2b). The recent expansion of ST21, particularly ST21C2, is remarkable. Although the mechanism(s) underlying these expansions are unknown at present, they may be related to the very successful global dissemination of ST21, which is the most predominant ST among O26:H11 clinical isolates [18, 24–26].

### Emergence of *stx2*-positive strains in multiple O26 : H11 lineages

Although the dissemination of *stx2*-positive ST29 strains is a global public-health concern [14, 18, 21, 27, 28, 30, 34], the dominant *stx* genotype of ST21 is *stx1*. However, a significant number of ST21 strains that harbour *stx2* alone or both *stx1* and *stx2* have also been detected [21, 25]. Our analysis revealed a highly scattered distribution of *stx2a* in both ST21 and ST29 (Fig. 1), indicative of a frequent gain (and probably also a loss) of Stx2a phages in multiple lineages of O26 : H11. Strains harbouring *stx2d* were found in the new French clonal lineage, which belongs to ST29C3, as reported elsewhere [27], and also in two different sublineages of ST21, suggesting that the infection of Stx2d phages is also occurring in multiple lineages of O26 : H11. In contrast, *stx1* was harboured by most ST21 strains, but found only in one strain in ST29. This finding suggests that *stx1* was acquired by a common ancestor of ST21 after its separation from ST29, and that it has been stably maintained in the ST21 lineage even though its deletion was observed in multiple sublineages.

In O157:H7, it was previously suggested that *stx2a* has relatively recently been acquired compared to other *stx* subtypes and that *stx2a* acquisition has occurred on multiple occasions [58]. The time-stamped phylogeny of O26 strains reconstituted in this study shows that *stx2*-positive strains emerged recently (mostly during the past 60 years) in most sublineages, particularly those in ST21C2 (Fig. 2), indicating that the gain of the Stx2a phage has recently and repeatedly occurred also in O26. This finding, together with the increased population size of ST21, suggests that this lineage could be the source of various highly virulent clones. In O157 : H7, the Stx2a production level is highly variable among strains, and we previously showed that the *stx2a*-encoding phage subtype is one of the determinants of the level of Stx2 production [61]. Among the O26 : H11 strains harbouring *stx2a*, the Stx2 production levels are also variable even if they belong to the same ST and clonal complex [18]. Therefore, fine and systematic analyses of the Stx2 production levels of each *stx2a*-positive O26 strain and of the subtypes of their Stx2a phages are necessary to monitor the emergence of highly virulent clones.

### T3SS effectors

**Fig. 3.**
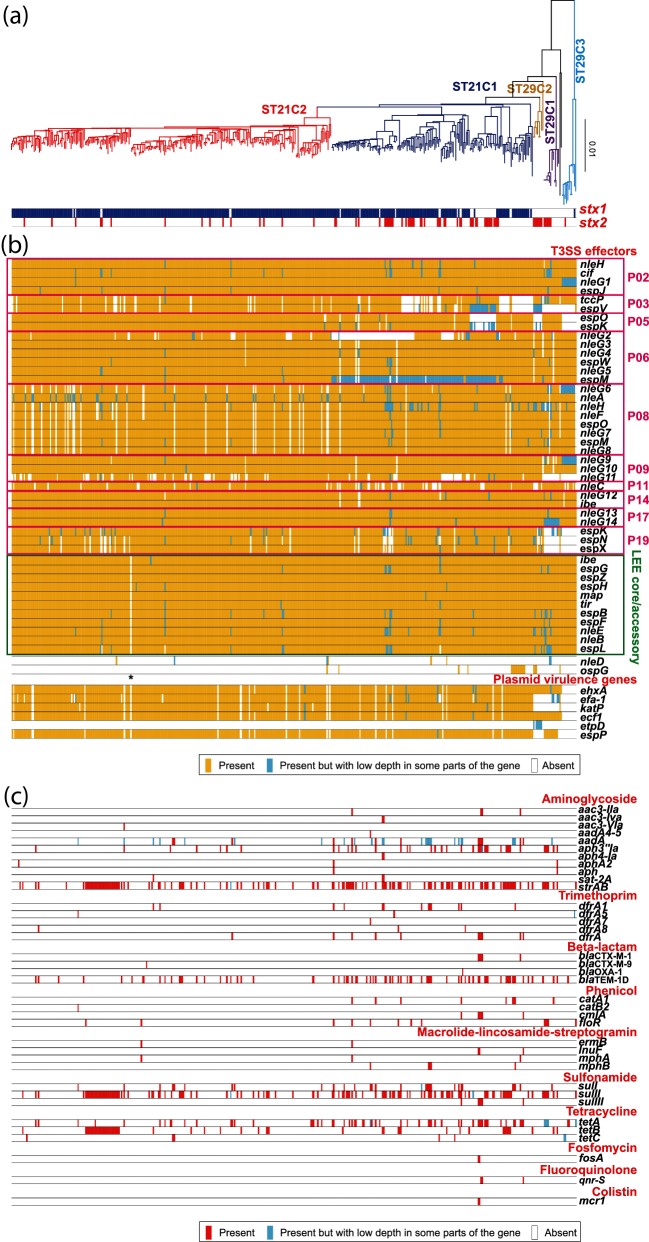
An ML tree of 429 O26 strains with heatmaps indicating the conservation of T3SS effector genes, plasmid-encoded VF genes and acquired AMR genes. (a) An ML tree was reconstructed based on the recombination-free SNPs identified on the core genome sequence of O26. Only strains in which Illumina read data were available were analysed. (b) Presence or absence of genes for T3SS effectors and plasmid-encoded VFs identified in the O26 reference strain 11368, which was determined using srst2 software. For the T3SS effector genes (*nleD* and *ospG*) and plasmid-encoded VF genes (*etpD* and *espP*) that are absent in strain 11368, the nucleotide sequences of ECs0850 (*nleD*), pO157_003 (*etpD*) and pO157_079 (*espP*) of O157 strain Sakai (BA000007) and ECO111_1634 (*ospG*) of O111 strain 11 128 (AP010960) were used as references. All of the locus_tag numbers of the T3SS effectors and plasmid-encoded VFs were used as references and are listed in Table S3. The names of the prophages (P02-P19) that encode the T3SS effectors in strain 11 368 are indicated. Genes predicted to be present, present but with low depth in some parts of the gene and absent are indicated by yellow, light blue and white, respectively. The strain TC6168 from which the LEE has been deleted is indicated by an asterisk. (c) Presence or absence of the acquired AMR genes was determined with srst2 software using ARGannot.r1.fasta as the resistance gene database. Genes predicted to be present, present but with low depth in some parts of the gene and absent are indicated by red, light blue and white, respectively.

We analysed the repertoire of T3SS effector genes using the srst2 program, a read mapping-based tool for gene content analysis [53]. Of the 181 strains with publicly available genome sequence data, raw Illumina read sequences were available for only 89 strains (Table S2). Thus, a total of 429 strains were used for this analysis (Fig. 3). Among the effector genes identified in the O26 : H11 reference strain 11368, which belongs to ST21C2, five [*espV*, *nleG* (ECO26_1978), *espK* (ECO26_3663), *espN* and *espX*] were only occasionally found in ST29, and two [*espO* and *espK* (ECO26_1525)] were absent in ST29C3, suggesting that these genes were not acquired by the common ancestors of ST29 and ST29C3, respectively. In contrast, the effector genes identified in the reference strain were well conserved in the ST21 strains, although sublineage-dependent deletions of effector genes were observed for several genes, such as *tccP*, *espV*, *espO*, *espK* and *nleG* (ECO26_1636), in ST21C1. Since *in vivo* functions of many of these effectors have not yet fully been elucidated [62], the clinical significance of this variation in T3SS effector profiles remains unclear. Numerous strain-specific deletions of effector genes were also observed in both ST21 and ST29. However, in many of these cases, genes found on the same prophage in the reference strain were coincidentally undetected (for example, P03, P08 and P19; Fig. 3, Table S5), suggesting that such prophages (or parts of them) have been deleted in a strain-specific manner.

Among the strains analysed, one strain (TC6168) did not contain the *eaeA* gene, an indicator of LEE. In this strain, all of the LEE-encoded effector genes were also absent, but most of the non-LEE effector genes were conserved, suggesting the recent deletion of the LEE element in this strain. Among the known T3SS effectors in LEE-positive *E. coli* strains, two families, *nleD* and *ospG,* are absent in the reference O26 : H11 strain, but both were present in a very limited number of strains (35 and 11 strains for *nleD* and *ospG,* respectively) (Fig. 3).

### Plasmid-encoded VFs

With the same strategy as that used for the T3SS effector genes, we analysed the conservation of four VF genes identified in the O26 virulence plasmid of the reference strain 11 368 (pO26_1) and two genes that were not encoded by pO26_1 but by the virulence plasmid of O157 : H7 (pO157). In all of the ST29C3 strains, these genes were completely absent (Fig. 3). Because most of the other genes on pO26_1 were also not detected in the ST29C3 strains (Fig. S2), this lineage most likely did not acquire any pO26_1-related plasmids. In the ST29C1 and ST29C2 strains, several pVF genes were detected (Fig. 3), as was previously observed [18, 21, 27, 28, 33]. In these strains, more pO26_1 genes, including the RepFIb replication protein encoding gene, were conserved, and the patterns of gene conservation were lineage-specific (Fig. S2). In ST21, all four VF genes on pO26_1 (Fig. 3), as well as other pO26_1 genes (Fig. S2), were well conserved even though pO26_1 appeared to be deleted in several strains. These findings suggest that a pO26_1-like plasmid was acquired by the common ancestor of the ST29C1, ST29C2 and ST21 lineages, and that lineage-specific diversification of the plasmid has taken place in each lineage. This scenario is supported by the fact that the overall topology of the core pO26_1 gene tree is consistent with that in the WG tree (Fig. S3).

Of the two genes originally identified in pO157, the *etpD* gene was detected only in the ST29C2 strains but at a low confidence (Fig. 3), suggesting that the gene was acquired specifically by this lineage and that its sequence has diverged significantly from that in pO157. The *espP* gene was detected in most of the ST21 strains and all of the ST29C1 strains. It is most likely that the *espP* gene is encoded by pO26_1-like plasmids and has been deleted from the plasmids of the reference strain and some of the other ST21 strains.

### Acquired AMR genes

The distribution of the acquired AMR genes in the O26 : H11 strains was also analysed by srst2 (Table S6). More than one-third of the strains (180 out of 429) contained at least one AMR gene, and a total of 37 AMR genes from 10 of the 13 categories in the ARGannot.r1.fasta database were detected in at least one of the tested strains (Fig. 3). Many different combinations of AMR genes were detected, but most were distributed in a strain or sublineage-specific manner, implying the frequent acquisition of different types of MGEs carrying these AMR genes.

In terms of the MGE-mediated acquisition of AMR genes, the distribution of pO26_2-related plasmids is intriguing. Plasmid pO26_2 was identified in the reference strain 11 368 and contained a gene encoding aminoglycoside-3′-phosphotransferase [APH(3′)] [6]. The *aph(3′)* gene was detected in relatively limited strains/sublineages, and their sequences often diverged from that of pO26_2. However, strains carrying many pO26_2 genes were distributed in all of the O26 : H11 lineages, including the most ancestral lineage ST29C3 (Fig. S4). In 30 % of the analysed strains (128 out of 429), more than half of the 84 genes on pO26_2 were detected. These conserved pO26_2 genes included genes for conjugal transfer. These findings indicate the wide distribution of pO26_2-related plasmids in O26, which may be involved in the acquisition of AMR genes by each strain/sublineage.

**Fig. 4.**
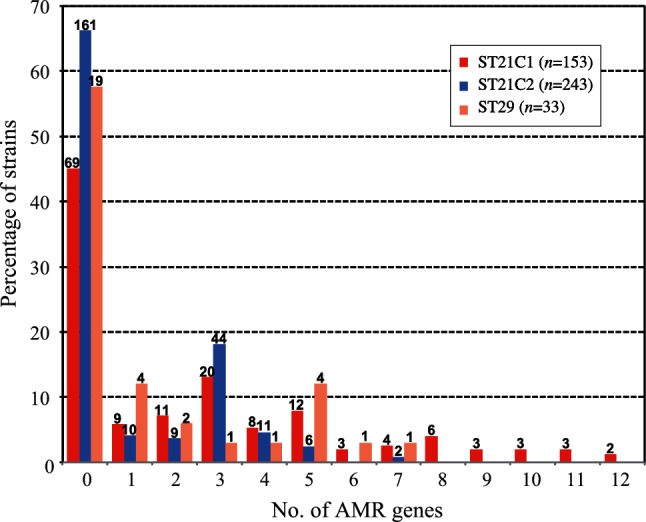
Number of acquired AMR genes identified in each O26 strain. The numbers of acquired AMR genes were counted in each strain, and the percentages of strains that contained the respective number of AMR genes are summarized and shown for ST29, ST21C1 and ST21C2. The numbers of strains that contained the respective number of AMR genes are indicated on the top of each bar.

Notably, the acquired AMR genes were more enriched in ST21C1 than in other lineages. Mean numbers of AMR genes per strain were 1.5 in ST29, 2.6 in ST21C1 and 1.0 in ST21C2. In addition, more than half of the ST21C1 strains (55 %) have acquired at least one AMR gene (Fig. 4). Although strains that have acquired multiple AMR genes were found in all of the lineages, those that had acquired 8 or more AMR genes were found only in ST21C1 (17 strains; 11 % of the ST21C1 strains examined), with two strains containing as many as 12 genes. The recently discovered plasmid-encoded colistin resistance gene (*mcr-1*) was also found in two closely related ST21C1 strains (Fig. 3) that were isolated from bovines in Japan and were hyper-multidrug-resistant strains carrying 11 AMR genes [63].

### Conclusion

Based on a large-scale genomic analysis, we have presented a global phylogenetic overview and evolutionary timescale for *E. coli* O26 : H11. The MRCA of O26 was estimated to be present approximately 415 years ago. ST21C1, one of the two sublineages of ST21, which is the most prevalent O26 : H11 lineage worldwide, emerged from one of the three known ST29 sublineages (ST29C2) approximately 213 years ago. The other ST21 lineage (ST21C2), which is now the most dominant lineage in Japan, emerged from ST21C1 approximately 95 years ago. Increases in population size occurred in the late 20th century for all of the O26 lineages, most remarkably for ST21C2. Bovine and human isolates were phylogenetically undistinguishable, at least in the ST21 lineage. Analysis of the distribution of *stx2*-positive strains revealed the recent and repeated acquisition of the *stx2* gene in multiple lineages of O26 : H11, both in ST21 and ST29. Other major VF genes of EHEC, T3SS effector genes and plasmid-encoded VF genes were well conserved in ST21 compared to ST29. In addition, more AMR genes have accumulated in the ST21C1 lineage. While more attention is now being paid to the emergence and dissemination of several ST29 clones that have acquired the *stx2* gene and, thus, are thought to be highly virulent, our findings indicate that there is also a risk that highly virulent clones could result from ST21. Therefore, longstanding routine surveillance of *stx2*-positive strains is also required for this lineage.

## Data bibliography

1. Ishijima N *et al*. GenBank/EMBL/DDBJ BioProject ID: PRJDB5136 (2017).
2. Public Health England. GenBank/EMBL/DDBJ BioProject ID: PRJNA315192 (2014).
3. Gastrointestinal Bacteria Reference Unit, Public Health England (GBRU). GenBank/EMBL/DDBJ BioProject ID: PRJNA259827 (2014).
4. Worley JN, Flores KA, Yang X *et al.* GenBank/EMBL/DDBJ BioProject ID: PRJNA230969 (2017).
5. Ferdous M, Zhou K, Mellmann A. GenBank/EMBL/DDBJ BioProject ID: PRJNA285020 (2015).
6. U.S. Food and Drug Administration. GenBank/EMBL/DDBJ BioProject ID: PRJNA242431, PRJNA175272, PRJNA129423 (2012).
7. Lindsey RL, *et al.* GenBank/EMBL/DDBJ BioProject ID: PRJNA218110 (2014).
8. Delannoy S *et al*. GenBank/EMBL/DDBJ BioProject ID: PRJNA284656 (2015).
9. Galia W *et al*. GenBank/EMBL/DDBJ accession number: CDLB01000000 (2014).
10. Lee JY, Chase B, Sohrabi A, Beck BJ. GenBank/EMBL/DDBJ accession number: AYOF01000000 (2003).
11. Hattori M, Ton H, Oshima K *et al*. GenBank/EMBL/DDBJ accession number: NC_013369 (2008).

## Supplementary Data

3967 Supplementary File 1
