# Supplementary material for: Population structure of Escherichia coli O26 : H11 with recent and repeated stx2 acquisition in multiple lineages
Source: Microb Genom. 2017 Nov 21;3(11):e000141. doi: 10.1099/mgen.0.000141 (PMC5729918; doi:10.1099/mgen.0.000141)
Supplement: Supplementary File 1 [file mgen-3-141-s001.pdf]

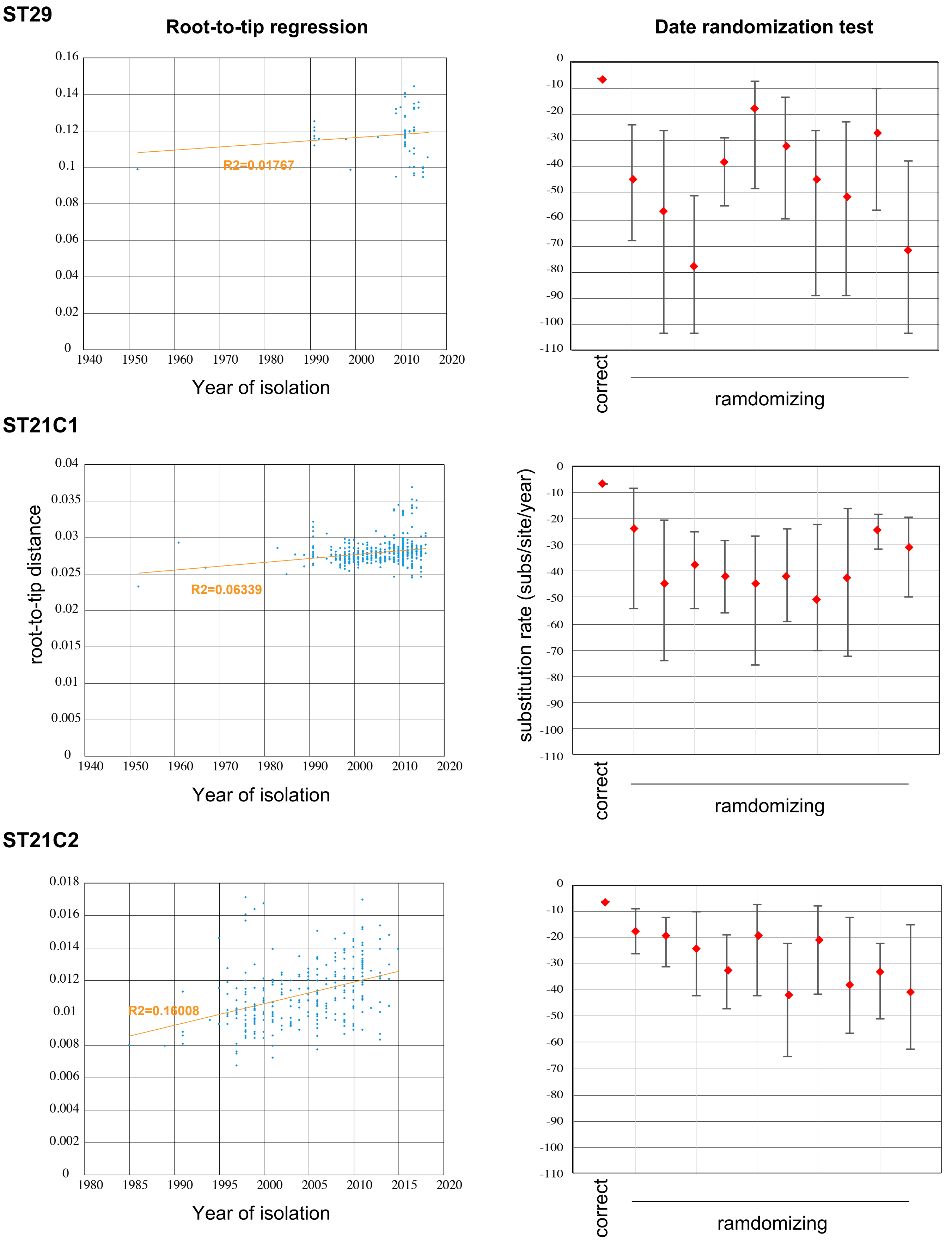

Figure S1. Results of the Root-to-tip regression and date randomization test

The ST29, ST21C1 and ST21C2 lineages were individually analyzed. On the left-hand side, the results of regression analyses of root-to-tip distance against sampling date. This analysis revealed a weak but positive correlation of genetic distance and sampling date in each lineage. On the right-hand side, the results of date randomization tests. The base substitution rates deduced with the correct sampling dates and those for ten date-randomized replicates are calculated in each lineage. Red circles represent the geometric mean and errors bars indicate the 95% credible interval. In each lineage, the mean substitution rates of randomized replicates and their 95% credible intervals were not within the 95% credible intervals deduced with the correct sampling times.

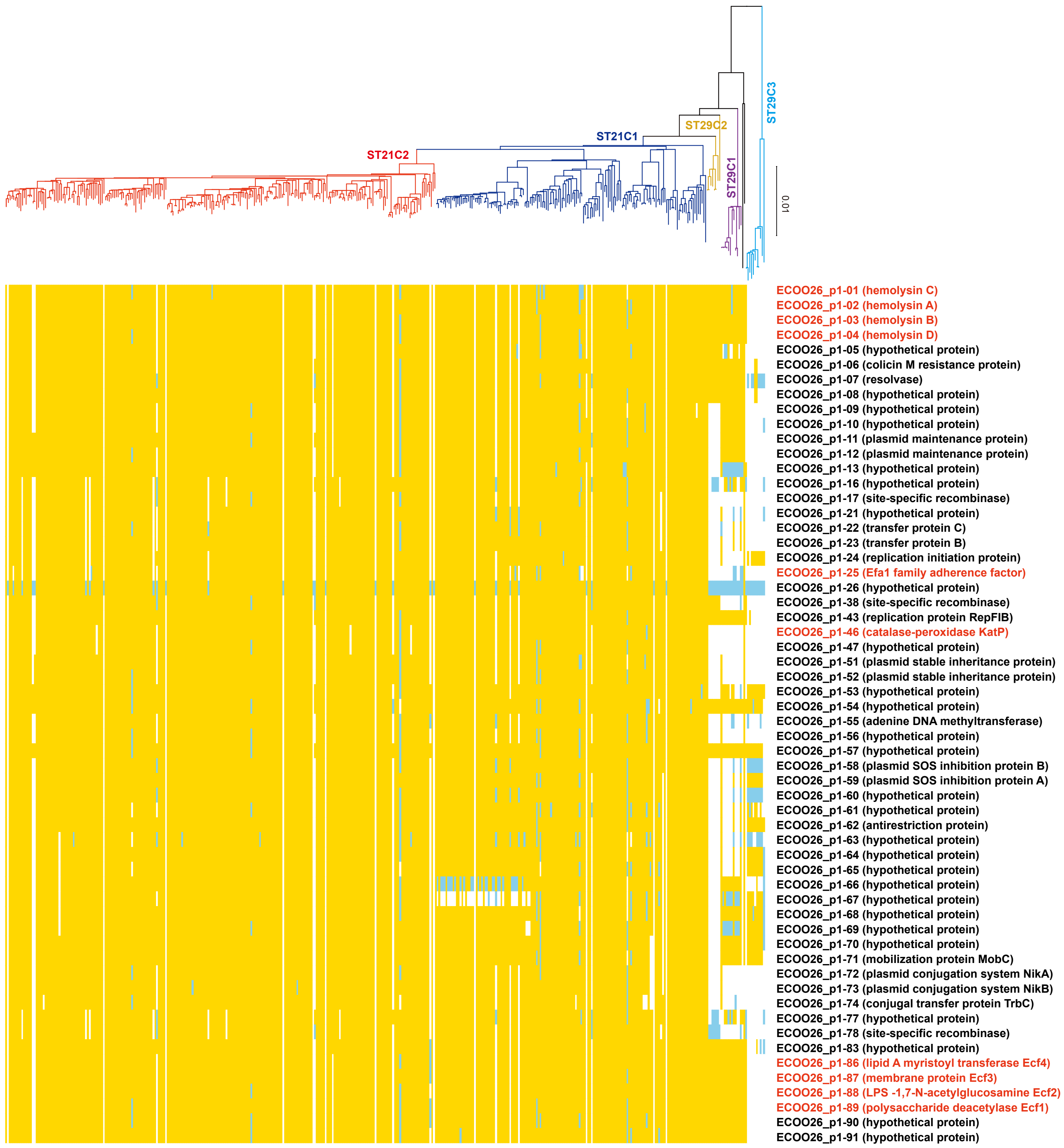

Figure S2. Conservation of the genes on pO26_1 in the 429 O26 strains.
Conservation of the genes on pO26_1 (AP010954), the virulence plasmid of strain 11368, among 429 O26 stains is shown with a ML tree of these strains. Yellow; present, light blue; present but with low depth in some parts of the gene in Illumina read mapping, no color; absent. The transposase genes on pO26_1 were excluded from the analysis. Virulence-related genes are indicated in red.

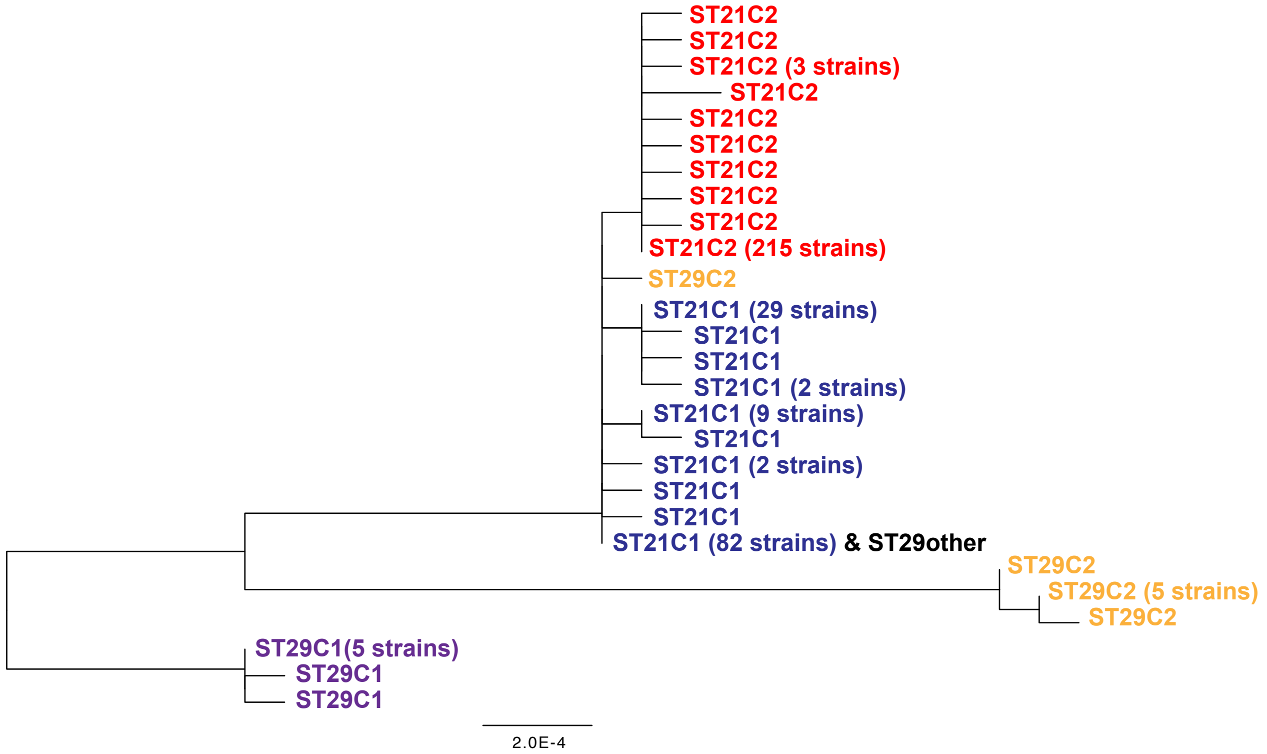

Figure S3. The putative phylogeny of O26 virulence plasmids
A neighbor-joining tree was constructed based on the SNPs identified in the 17 core genes of the O26 virulence plasmid, which were conserved in most O26 strains belonging to the four major lineages (ST29C1, ST29C2, ST29C1, and ST21C2) and a minor ST29 lineage. SNPs were identified by read mapping to the O26 virulence plasmid (pO26_1) of the reference strain. As we excluded all strains that lack any of the 17 genes from this analysis, a total of 370 strains (7 strains of ST29C1, 7 of ST29C2, 1 of the minor ST29 lineages, 129 of ST21C2 and 226 of ST21C2) were analyzed. Although the resolution is much lower, the overall topology is consistent to that in the whole gene tree (Fig. 3 in the main text), except for one ST29C2 strain and the ST29 strain belonging to a minor lineage (indicated as "ST29other" in the tree). As recombinogenic SNP sites were not excluded in this analysis, their phylogenetic positions may be resulted from some recombination events occurred in these strains (or sublineages leading to these strains). Maximum likelihood reconstitution of the phylogeny yielded the same result. The locus_tag numbers of the 17 plasmid core genes are as follows: ECO26_p1-01, ECO26_p1-02, ECO26_p1-03, ECO26_p1-04, ECO26_p1-06, ECO26_p1-07, ECO26_p1-08, ECO26_p1-43, ECO26_p1-54, ECO26_p1-57, ECO26_p1-83, ECO26_p1-86, ECO26_p1-87, ECO26_p1-88, ECO26_p1-89, ECO26_p1-90, ECO26_p1-91.

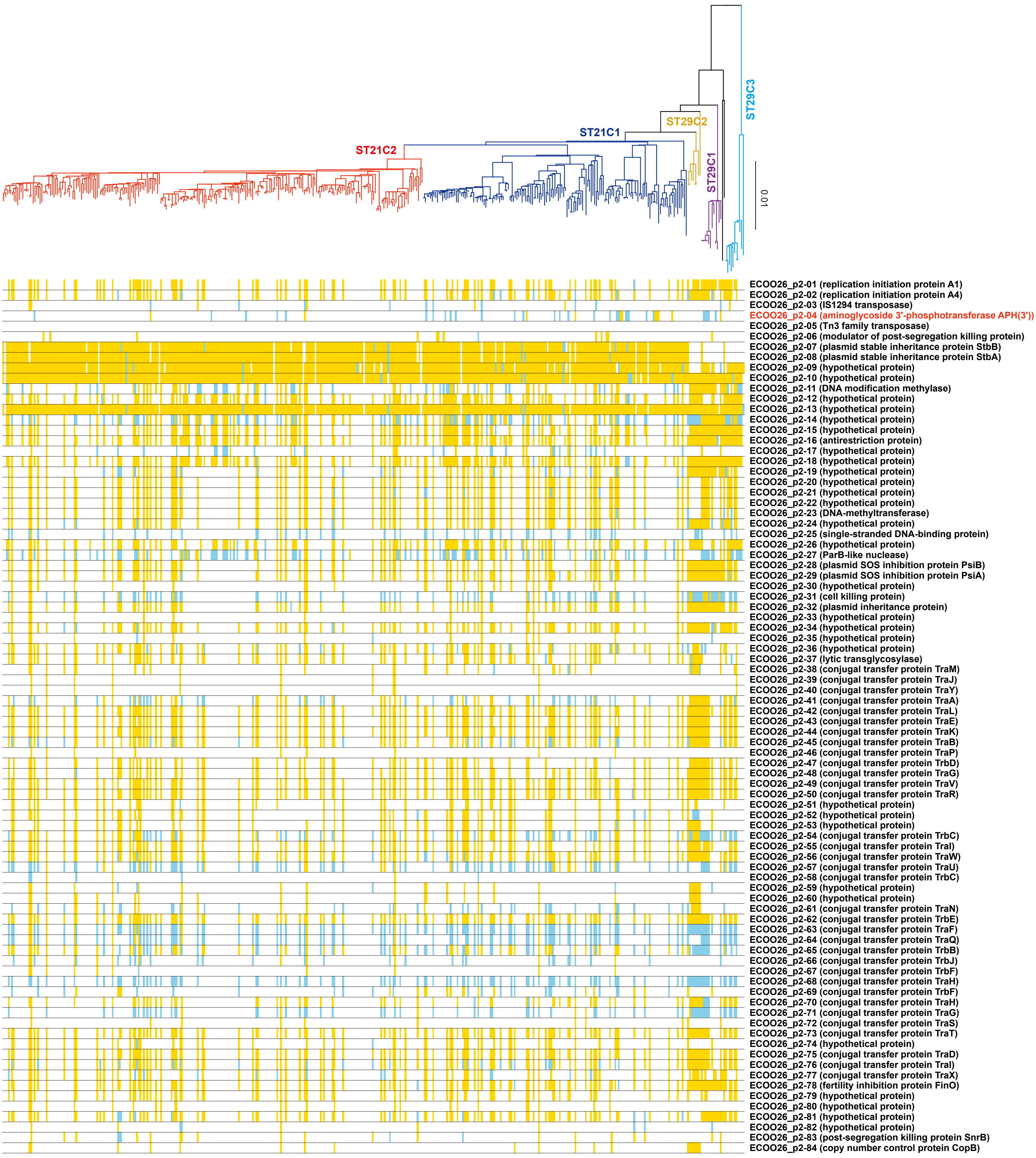

Figure S4. Conservation of the genes on pO26_2in the 429 O26 strains

Conservation of the genes on pO26_2 (AP010955), an AMR plasmid found in strain 11368, among 429 O26 stains is shown with a ML tree of these strains. Yellow; present, light blue; present but with low depth in some parts of the gene in Illumina read mapping, no color; absent. The gene for aminoglycoside-3'-phosphotransferase (APH(3') is indicated in red.

Table S1. Characteristics of O26 strains sequenced in this study

ID	strain name	year of isolation	source	country	ST	lineage	plasmid gene profile	*stx1*	*stx2*	intimin (eaeA) subtype	age	gender	diarrhea	bloody diarrhea	HUS	no. of scaffold	longest scaffold length	total scaffold length	average coverage	accession no.	
Aki01	EC5337	2001	bovine	Japan	ST21	ST21C2	*ehxA+/katP+/espP+/etpD-*	*stx1a*	-	beta1	NS	NS	NS	NS	NS	284	180161	5637765	30	DRR103385	BEIW01000001-BEIW01000284
Aki02	EC15670	2013	bovine	Japan	ST21	ST21C1	*ehxA+/katP+/espP+/etpD-*	-	-	beta1	NS	NS	NS	NS	NS	288	299472	5633754	37	DRR103386	BEIX01000001-BEIX01000288
Aki03	EC15671	2013	bovine	Japan	ST29	ST29C1	*ehxA+/katP-/espP+/etpD-*	-	-	beta1	NS	NS	NS	NS	NS	227	266404	5358002	38	DRR103387	BEIY01000001-BEIY01000227
Aki04	EC15674	2013	bovine	Japan	ST21	ST21C2	*ehxA+/katP+/espP+/etpD-*	*stx1a*	-	beta1	NS	NS	NS	NS	NS	259	355230	5532084	41	DRR103388	BEIZ01000001-BEIZ01000259
Aki05	EC15718	2013	bovine	Japan	ST21	ST21C2	*ehxA+/katP+/espP+/etpD-*	*stx1a*	-	beta1	NS	NS	NS	NS	NS	250	355107	5529073	50	DRR103389	BEJA01000001-BEJA01000250
Aki06	EC15720	2013	bovine	Japan	ST29	ST29C1	*ehxA+/katP-/espP+/etpD-*	-	-	beta1	NS	NS	NS	NS	NS	219	333708	5365930	42	DRR103390	BEJB01000001-BEJB01000219
Aki07	EC15802	2013	ovine	Japan	ST21	ST21C2	*ehxA+/katP+/espP+/etpD-*	*stx1a*	-	beta1	NS	NS	NS	NS	NS	250	284624	5555083	56	DRR103391	BEJC01000001-BEJC01000250
B02	EH031	1989	human	Belgium	ST21	ST21C1	*ehxA-/katP-/espP-/etpD-*	*stx1a*	-	beta1	NS	NS	NS	NS	NS	326	256798	5496664	61	DRR103392	BEJD01000001-BEJD01000326
B04	EH322	1996	human	Belgium	ST21	ST21C1	*ehxA+/katP+/espP+/etpD-*	*stx1a*	-	beta1	NS	NS	NS	NS	NS	389	250469	5678689	113	DRR103393	BEJE01000001-BEJE01000389
B07	TC3108	1991	bovine	USA	ST21	ST21C2	*ehxA-/katP-/espP-/etpD-*	*stx1a*	-	beta1	NS	NS	NS	NS	NS	313	256418	5344185	68	DRR103394	BEJF01000001-BEJF01000313
B09	TC3117	1991	bovine	USA	ST21	ST21C2	*ehxA+/katP+/espP+/etpD-*	*stx1a*	-	beta1	NS	NS	NS	NS	NS	365	230533	5543229	80	DRR103395	BEJG01000001-BEJG01000365
B10	TC3145	1991	bovine	USA	ST29	ST29C1	*ehxA+/katP-/espP+/etpD-*	-	-	beta1	NS	NS	NS	NS	NS	360	237682	5583152	35	DRR103396	BEJH01000001-BEJH01000360
B11	TC3180	1991	bovine	USA	ST21	ST21C1	*ehxA+/katP+/espP+/etpD-*	*stx1a*	-	beta1	NS	NS	NS	NS	NS	351	255167	5547691	74	DRR103397	BEJI01000001-BEJI01000351
B12	TC3269	1991	bovine	USA	ST21	ST21C1	*ehxA+/katP+/espP+/etpD-*	*stx1a*	-	beta1	NS	NS	NS	NS	NS	350	324563	5665136	81	DRR103398	BEJJ01000001-BEJJ01000350
B13	TC3273	1991	bovine	USA	ST21	ST21C1	*ehxA+/katP+/espP+/etpD-*	*stx1a*	-	beta1	NS	NS	NS	NS	NS	280	255425	5526894	72	DRR103399	BEJK01000001-BEJK01000280
B14	TC3302	1991	bovine	USA	ST21	ST21C1	*ehxA+/katP+/espP+/etpD-*	*stx1a*	-	beta1	NS	NS	NS	NS	NS	379	244296	5759022	69	DRR103400	BEJL01000001-BEJL01000379
B15	TC3305	1991	bovine	USA	ST21	ST21C1	*ehxA+/katP+/espP+/etpD-*	*stx1a*	-	beta1	NS	NS	NS	NS	NS	353	244415	5719178	88	DRR103401	BEJM01000001-BEJM01000353
B16	TC3375	1991	bovine	USA	ST21	ST21C1	*ehxA+/katP+/espP+/etpD-*	*stx1a*	-	beta1	NS	NS	NS	NS	NS	352	249647	5663334	66	DRR103402	BEJN01000001-BEJN01000352
B17	TC3380	1991	bovine	USA	ST21	ST21C1	*ehxA+/katP+/espP+/etpD-*	*stx1a*	-	beta1	NS	NS	NS	NS	NS	343	253513	5704602	70	DRR103403	BEJO01000001-BEJO01000343
B18	TC3486	1991	bovine	USA	ST29	ST29C1	*ehxA+/katP-/espP+/etpD-*	-	-	beta1	NS	NS	NS	NS	NS	364	279020	5597575	94	DRR103404	BEJP01000001-BEJP01000364
B20	TC3630	1991	bovine	USA	ST21	ST21C2	*ehxA+/katP+/espP+/etpD-*	*stx1a*	-	beta1	NS	NS	NS	NS	NS	311	262064	5577712	71	DRR103405	BEJQ01000001-BEJQ01000311
B23	TC3656	1991	bovine	USA	ST21	ST21C2	*ehxA+/katP+/espP+/etpD-*	*stx1a*	-	beta1	NS	NS	NS	NS	NS	303	230438	5555861	92	DRR103406	BEJR01000001-BEJR01000303
B25	TC3748	1991	bovine	USA	ST29	ST29C1	*ehxA+/katP-/espP+/etpD-*	-	-	beta1	NS	NS	NS	NS	NS	254	267143	5245421	89	DRR103407	BEJS01000001-BEJS01000254
B26	TC4219	1991	bovine	USA	ST21	ST21C1	*ehxA+/katP+/espP+/etpD-*	-	-	beta1	NS	NS	NS	NS	NS	319	269772	5795580	105	DRR103408	BEJT01000001-BEJT01000319
B28	TC6165	1961	human	USA	ST21	ST21C1	*ehxA+/katP+/espP+/etpD-*	*stx1a*	-	beta1	NS	NS	NS	NS	NS	286	273357	5464808	87	DRR103409	BEJU01000001-BEJU01000286
B29	TC6168	1997	human	USA	ST21	ST21C2	*ehxA-/katP-/espP-/etpD-*	*stx1a*	-	negative	NS	NS	NS	NS	NS	262	249979	5321497	62	DRR103410	BEJV01000001-BEJV01000262
B30	TC6169	1989	bovine	USA	ST21	ST21C2	*ehxA-/katP-/espP-/etpD-*	*stx1a*	-	beta1	NS	NS	NS	NS	NS	281	230862	5388833	89	DRR103411	BEJW01000001-BEJW01000281
B31	TC3848	1991	bovine	USA	ST29	ST29C1	*ehxA+/katP-/espP+/etpD-*	-	-	beta1	NS	NS	NS	NS	NS	239	265647	5481870	79	DRR103412	BEJX01000001-BEJX01000239
B33	TC6167	1952	human	Swiss	ST29	ST29C3	*ehxA-/katP-/espP-/etpD-*	-	-	beta1	NS	NS	NS	NS	NS	213	250419	5309810	64	DRR103413	BEJY01000001-BEJY01000213
B35	02/113	2002	human	France	ST21	ST21C2	*ehxA+/katP+/espP+/etpD-*	*stx1a*	-	beta1	NS	NS	NS	NS	NS	289	230546	5505243	97	DRR103414	BEJZ01000001-BEJZ01000289
B36	99/109	1999	human	France	ST29	ST29C2	*ehxA+/katP-/espP-/etpD-*	-	*stx2a*	beta1	NS	NS	NS	NS	NS	247	334255	5485044	131	DRR103415	BEKA01000001-BEKA01000247
B37	03/139	2003	human	France	new	ST21C1	*ehxA+/katP+/espP+/etpD-*	*stx1a*	-	beta1	NS	NS	NS	NS	NS	339	250030	5617267	92	DRR103416	BEKB01000001-BEKB01000339
B40	333KH91	1991	bovine	Netherlands	ST29	ST29C3	*ehxA-/katP-/espP-/etpD-*	-	-	beta1	NS	NS	NS	NS	NS	225	250441	5497494	124	DRR103417	BEKC01000001-BEKC01000225
B41	631KH91	1991	bovine	Belgium	ST29	ST29C3	*ehxA-/katP-/espP-/etpD-*	-	-	beta1	NS	NS	NS	NS	NS	240	296628	5570667	106	DRR103418	BEKD01000001-BEKD01000240
B42	H19	1967	human	UK	ST21	ST21C1	*ehxA+/katP+/espP+/etpD-*	*stx1a*	-	beta1	NS	NS	NS	NS	NS	287	256879	5555471	110	DRR103419	BEKE01000001-BEKE01000287
B43	11.2	2008	bovine	Belgium	ST1705	ST21C1	*ehxA+/katP+/espP+/etpD-*	*stx1a*	-	beta1	NS	NS	NS	NS	NS	362	244644	5648204	87	DRR103420	BEKF01000001-BEKF01000362
B44	20.1	2008	bovine	Belgium	ST21	ST21C1	*ehxA-/katP-/espP-/etpD-*	*stx1a*	-	beta1	NS	NS	NS	NS	NS	309	250638	5493848	132	DRR103421	BEKG01000001-BEKG01000309
B45	ED80	1992	bovine	Italy	ST29	ST29C3	*ehxA-/katP-/espP-/etpD-*	-	-	beta1	NS	NS	NS	NS	NS	252	250426	5492359	98	DRR103422	BEKH01000001-BEKH01000252
B46	EH182	1994	human	Belgium	ST21	ST21C2	*ehxA+/katP+/espP+/etpD-*	*stx1a*	-	beta1	NS	NS	NS	NS	NS	299	245458	5609553	95	DRR103423	BEKI01000001-BEKI01000299
B47	EH193	1994	human	Belgium	ST21	ST21C1	*ehxA-/katP-/espP-/etpD-*	-	-	beta1	NS	NS	NS	NS	NS	255	245013	5330202	115	DRR103424	BEKJ01000001-BEKJ01000255
B48	357S89	1987	bovine	Belgium	ST21	ST21C1	*ehxA+/katP+/espP+/etpD-*	*stx1a*	-	beta1	NS	NS	NS	NS	NS	335	269348	5524055	125	DRR103425	BEKK01000001-BEKK01000335
BB01	11KH63	2011	bovine	Belgium	ST21	ST21C1	*ehxA+/katP+/espP+/etpD-*	*stx1a*	-	beta1	NS	NS	NS	NS	NS	271	230508	5675140	37	DRR103426	BEKL01000001-BEKL01000271
BB02	11KH245	2011	bovine	Belgium	ST21	ST21C1	*ehxA+/katP+/espP+/etpD-*	*stx1a*	-	beta1	NS	NS	NS	NS	NS	281	224099	5864077	50	DRR103427	BEKM01000001-BEKM01000281
BB03	11KH263	2011	bovine	Belgium	ST21	ST21C1	*ehxA+/katP+/espP+/etpD-*	*stx1a*	*stx2a*	beta1	NS	NS	NS	NS	NS	360	250432	5749982	74	DRR103428	BEKN01000001-BEKN01000360
BB04	12KH23	2012	bovine	Belgium	ST21	ST21C1	*ehxA+/katP+/espP+/etpD-*	*stx1a*	-	beta1	NS	NS	NS	NS	NS	341	222775	5804445	75	DRR103429	BEKO01000001-BEKO01000341
BK01	E0437	2011	bovine	Japan	ST21	ST21C2	*ehxA+/katP+/espP+/etpD-*	*stx1a*	-	beta1	NS	NS	NS	NS	NS	331	354668	5732604	87	DRR103430	BEKP01000001-BEKP01000331
BK03	E0439	2011	bovine	Japan	ST21	ST21C1	*ehxA+/katP+/espP+/etpD-*	*stx1a*	-	beta1	NS	NS	NS	NS	NS	316	230637	5731277	99	DRR103431	BEKQ01000001-BEKQ01000316
BK04	E0440	2011	bovine	Japan	ST21	ST21C1	*ehxA+/katP+/espP+/etpD-*	-	-	beta1	NS	NS	NS	NS	NS	262	256594	5532904	85	DRR103432	BEKR01000001-BEKR01000262
BK05	E0441	2011	bovine	Japan	ST21	ST21C1	*ehxA+/katP+/espP+/etpD-*	*stx1a*	-	beta1	NS	NS	NS	NS	NS	320	230785	5634430	102	DRR103433	BEKS01000001-BEKS01000320
BK07	E0443	2011	bovine	Japan	ST21	ST21C2	*ehxA+/katP+/espP+/etpD-*	*stx1a*	-	beta1	NS	NS	NS	NS	NS	341	277171	5733854	150	DRR103434	BEKT01000001-BEKT01000341
BK08	E0444	2011	bovine	Japan	ST21	ST21C2	*ehxA+/katP+/espP+/etpD-*	*stx1a*	-	beta1	NS	NS	NS	NS	NS	322	354796	5719538	54	DRR103435	BEKU01000001-BEKU01000322
BK09	E0445	2011	bovine	Japan	ST21	ST21C2	*ehxA+/katP-/espP+/etpD-*	*stx1a*	-	beta1	NS	NS	NS	NS	NS	320	277193	5696101	141	DRR103436	BEKV01000001-BEKV01000320
BK10	E0446	2011	bovine	Japan	ST21	ST21C2	*ehxA+/katP+/espP+/etpD-*	*stx1a*	-	beta1	NS	NS	NS	NS	NS	286	247340	5502646	80	DRR103437	BEKW01000001-BEKW01000286
BK11	E0447	2011	bovine	Japan	ST21	ST21C1	*ehxA+/katP+/espP+/etpD-*	-	-	beta1	NS	NS	NS	NS	NS	278	286045	5574050	102	DRR103438	BEKX01000001-BEKX01000278
BK13	E0482	2009	bovine	Japan	ST21	ST21C2	*ehxA+/katP+/espP+/etpD-*	*stx1a*	-	beta1	NS	NS	NS	NS	NS	272	297356	5511644	126	DRR103439	BEKY01000001-BEKY01000272
BK23	E0497	2009	bovine	Japan	ST21	ST21C2	*ehxA+/katP+/espP+/etpD-*	*stx1a*	-	beta1	NS	NS	NS	NS	NS	322	354924	5761829	144	DRR103440	BEKZ01000001-BEKZ01000322
F01	97E12-1	1997	human	Japan	ST21	ST21C1	*ehxA+/katP+/espP+/etpD-*	*stx1a*	-	beta1	NS	NS	+	-	-	397	230263	5571648	86	DRR103441	BELA01000001-BELA01000397
F02	97E16	1997	human	Japan	ST21	ST21C2	*ehxA+/katP+/espP+/etpD-*	*stx1a*	-	beta1	NS	NS	+	+	-	318	277052	5483592	172	DRR103442	BELB01000001-BELB01000318
F03	98E4	1998	human	Japan	ST21	ST21C2	*ehxA+/katP+/espP+/etpD-*	*stx1a*	-	beta1	NS	NS	+	-	-	281	232634	5305244	163	DRR103443	BELC01000001-BELC01000281
F04	98E11	1998	human	Japan	ST21	ST21C2	*ehxA+/katP+/espP+/etpD-*	*stx1a*	-	beta1	NS	NS	+	+	-	364	273270	5624198	112	DRR103444	BELD01000001-BELD01000364
F05	99E46	1999	human	Japan	ST21	ST21C2	*ehxA+/katP+/espP+/etpD-*	*stx1a*	-	beta1	NS	NS	+	-	-	312	281894	5564670	130	DRR103445	BELE01000001-BELE01000312
F06	99E13	1999	human	Japan	ST21	ST21C1	*ehxA+/katP+/espP+/etpD-*	*stx1a*	*stx2a*	beta1	NS	NS	+	-	-	354	230785	5573574	117	DRR103446	BELF01000001-BELF01000354

F07	99E24	1999	human	Japan	ST21	ST21C2	*ehxA+/katP+/espP+/etpD-*	*stx1a*	-	beta1	NS	NS	+	+	-	307	230875	5529272	122	DRR103447	BELG01000001-BELG01000307
F08	00E021	2000	human	Japan	ST21	ST21C2	*ehxA+/katP+/espP+/etpD-*	*stx1a*	-	beta1	NS	NS	+	-	-	358	250565	5528728	101	DRR103448	BELH01000001-BELH01000358
F09	00E027	2000	human	Japan	ST21	ST21C1	*ehxA+/katP+/espP+/etpD-*	*stx1a*	-	beta1	NS	NS	+	-	-	397	244501	5573903	133	DRR103449	BELI01000001-BELI01000397
F10	00E107	2000	human	Japan	ST21	ST21C2	*ehxA-/katP-/espP-/etpD-*	*stx1a*	-	beta1	NS	NS	+	+	-	346	255915	5425984	107	DRR103450	BELJ01000001-BELJ01000346
F11	00E110	2000	human	Japan	ST21	ST21C2	*ehxA+/katP+/espP+/etpD-*	*stx1a*	-	beta1	NS	NS	+	+	-	362	250639	5559642	97	DRR103451	BELK01000001-BELK01000362
F12	02E003	2002	human	Japan	ST21	ST21C2	*ehxA+/katP+/espP+/etpD-*	*stx1a*	-	beta1	NS	NS	+	+	-	284	250213	5444251	96	DRR103452	BELL01000001-BELL01000284
F14	02E022	2002	human	Japan	ST21	ST21C2	*ehxA+/katP+/espP+/etpD-*	*stx1a*	-	beta1	NS	NS	+	+	-	324	234959	5603009	89	DRR103453	BELM01000001-BELM01000324
F15	02E049	2002	human	Japan	ST21	ST21C1	*ehxA+/katP+/espP+/etpD-*	*stx1a*	-	beta1	NS	NS	+	+	-	395	244377	5584028	123	DRR103454	BELN01000001-BELN01000395
F16	02E052	2002	human	Japan	ST21	ST21C2	*ehxA+/katP+/espP+/etpD-*	*stx1a*	-	beta1	NS	NS	+	-	-	343	297612	5514326	124	DRR103455	BELO01000001-BELO01000343
F17	02E060	2002	human	Japan	ST21	ST21C2	*ehxA+/katP+/espP+/etpD-*	*stx1a*	*stx2a*	beta1	NS	NS	+	-	-	371	267414	5520183	161	DRR103456	BELP01000001-BELP01000371
F18	03E003	2003	human	Japan	ST21	ST21C2	*ehxA+/katP+/espP+/etpD-*	*stx1a*	-	beta1	NS	NS	+	+	-	269	244480	5559028	123	DRR103457	BELQ01000001-BELQ01000269
F19	03E016	2003	human	Japan	ST21	ST21C1	*ehxA+/katP+/espP+/etpD-*	*stx1a*	-	beta1	NS	NS	+	-	-	353	230692	5671236	59	DRR103458	BELR01000001-BELR01000353
F20	04E002	2004	human	Japan	ST21	ST21C2	*ehxA+/katP+/espP+/etpD-*	*stx1a*	-	beta1	NS	NS	+	+	-	370	234960	5545131	81	DRR103459	BELS01000001-BELS01000370
F21	04E091	2004	human	Japan	ST21	ST21C2	*ehxA+/katP+/espP+/etpD-*	*stx1a*	-	beta1	NS	NS	+	—	-	333	250168	5448490	79	DRR103460	BELT01000001-BELT01000333
F22	05E031	2005	human	Japan	ST21	ST21C1	*ehxA+/katP+/espP+/etpD-*	*stx1a*	-	beta1	NS	NS	+	-	-	345	283110	5577792	110	DRR103461	BELU01000001-BELU01000345
F23	05E035	2005	human	Japan	ST21	ST21C2	*ehxA+/katP+/espP+/etpD-*	*stx1a*	-	beta1	NS	NS	+	—	-	325	251181	5454247	77	DRR103462	BELV01000001-BELV01000325
F24	06E003	2006	human	Japan	ST21	ST21C2	*ehxA+/katP+/espP+/etpD-*	*stx1a*	-	beta1	NS	NS	+	+	-	300	235042	5427307	93	DRR103463	BELW01000001-BELW01000300
F25	06E028	2006	human	Japan	ST21	ST21C2	*ehxA+/katP+/espP+/etpD-*	*stx1a*	-	beta1	NS	NS	+	-	-	316	254068	5489759	78	DRR103464	BELX01000001-BELX01000316
F26	06E048	2006	human	Japan	ST21	ST21C2	*ehxA+/katP+/espP+/etpD-*	*stx1a*	-	beta1	NS	NS	+	+	-	331	256412	5562637	75	DRR103465	BELY01000001-BELY01000331
F28	06E065	2006	human	Japan	ST21	ST21C2	*ehxA+/katP+/espP+/etpD-*	*stx1a*	-	beta1	NS	NS	+	-	-	313	354870	5539796	67	DRR103466	BELZ01000001-BELZ01000313
F29	06E070	2006	human	Japan	ST21	ST21C2	*ehxA+/katP+/espP+/etpD-*	*stx1a*	-	beta1	NS	NS	+	-	-	315	355472	5474608	120	DRR103467	BEMA01000001-BEMA01000315
F30	07E043	2007	human	Japan	ST21	ST21C2	*ehxA+/katP+/espP+/etpD-*	*stx1a*	-	beta1	NS	NS	+	-	-	298	231165	5521513	108	DRR103468	BEMB01000001-BEMB01000298
F31	07E063	2007	human	Japan	ST21	ST21C1	*ehxA+/katP+/espP+/etpD-*	*stx1a*	-	beta1	NS	NS	+	+	-	351	286576	5588006	117	DRR103469	BEMC01000001-BEMC01000351
F32	07E069	2007	human	Japan	ST21	ST21C2	*ehxA+/katP+/espP+/etpD-*	*stx1a*	-	beta1	NS	NS	-	+	-	326	354806	5544499	97	DRR103470	BEMD01000001-BEMD01000326
F33	08E004	2008	human	Japan	ST21	ST21C2	*ehxA+/katP+/espP+/etpD-*	*stx1a*	-	beta1	NS	NS	+	-	-	330	354913	5581332	109	DRR103471	BEME01000001-BEME01000330
F34	08E010	2008	human	Japan	ST21	ST21C2	*ehxA+/katP+/espP+/etpD-*	*stx1a*	-	beta1	NS	NS	+	+	-	309	250377	5446911	110	DRR103472	BEMF01000001-BEMF01000309
F35	08E014	2008	human	Japan	ST21	ST21C2	*ehxA+/katP+/espP+/etpD-*	*stx1a*	-	beta1	NS	NS	+	+	-	304	250219	5550168	115	DRR103473	BEMG01000001-BEMG01000304
F36	08E018	2008	human	Japan	ST21	ST21C2	*ehxA+/katP+/espP+/etpD-*	*stx1a*	-	beta1	NS	NS	+	-	-	374	273292	5719853	90	DRR103474	BEMH01000001-BEMH01000374
F37	08E101	2008	human	Japan	ST21	ST21C2	*ehxA+/katP+/espP+/etpD-*	*stx1a*	-	beta1	NS	NS	+	-	-	377	230043	5472308	140	DRR103475	BEMI01000001-BEMI01000377
F39	08E127	2008	human	Japan	ST21	ST21C2	*ehxA+/katP+/espP+/etpD-*	*stx1a*	-	beta1	NS	NS	+	-	-	341	230774	5530544	114	DRR103476	BEMJ01000001-BEMJ01000341
F40	09E030	2009	human	Japan	ST21	ST21C2	*ehxA+/katP+/espP+/etpD-*	*stx1a*	-	beta1	NS	NS	+	+	-	300	230975	5542461	109	DRR103477	BEMK01000001-BEMK01000300
F41	09E040	2009	human	Japan	ST21	ST21C2	*ehxA+/katP+/espP+/etpD-*	*stx1a*	-	beta1	NS	NS	+	-	-	374	256798	5631614	125	DRR103478	BEML01000001-BEML01000374
F42	09E107	2009	human	Japan	ST21	ST21C2	*ehxA+/katP+/espP+/etpD-*	*stx1a*	-	beta1	NS	NS	+	-	-	313	354849	5620390	61	DRR103479	BEMM01000001-BEMM01000313
F43	09E131	2009	human	Japan	ST21	ST21C2	*ehxA+/katP+/espP+/etpD-*	*stx1a*	-	beta1	NS	NS	+	+	-	334	230426	5476545	73	DRR103480	BEMN01000001-BEMN01000334
F44	10E005	2010	human	Japan	ST21	ST21C2	*ehxA+/katP+/espP+/etpD-*	*stx1a*	-	beta1	NS	NS	+	+	-	354	254233	5683151	81	DRR103481	BEMO01000001-BEMO01000354
F46	11E033	2011	human	Japan	ST21	ST21C2	*ehxA+/katP+/espP+/etpD-*	*stx1a*	-	beta1	NS	NS	+	+	-	329	254481	5455087	115	DRR103482	BEMP01000001-BEMP01000329
F47	11E044	2011	human	Japan	ST21	ST21C2	*ehxA+/katP+/espP+/etpD-*	*stx1a*	-	beta1	NS	NS	+	+	-	344	230302	5712559	65	DRR103483	BEMQ01000001-BEMQ01000344
F48	11E059	2011	human	Japan	ST21	ST21C2	*ehxA+/katP+/espP+/etpD-*	*stx1a*	-	beta1	NS	NS	+	-	-	375	267392	5600400	72	DRR103484	BEMR01000001-BEMR01000375
M01	10002	1994	human	Japan	ST21	ST21C1	*ehxA+/katP+/espP+/etpD-*	*stx1a*	-	beta1	NS	NS	+	-	-	302	318647	5510210	124	DRR103485	BEMS01000001-BEMS01000302
M02	10010	1995	human	Japan	ST21	ST21C2	*ehxA+/katP+/espP+/etpD-*	*stx1a*	-	beta1	NS	NS	+	-	-	285	230357	5667076	122	DRR103486	BEMT01000001-BEMT01000285
M03	10012	1995	human	Japan	ST21	ST21C2	*ehxA+/katP+/espP+/etpD-*	*stx1a*	-	beta1	NS	NS	+	+	-	286	256544	5589941	121	DRR103487	BEMU01000001-BEMU01000286
M04	10014	1996	human	Japan	ST21	ST21C2	*ehxA+/katP+/espP+/etpD-*	*stx1a*	-	beta1	NS	NS	+	+	-	276	354743	5678724	117	DRR103488	BEMV01000001-BEMV01000276
M05	10019	1996	human	Japan	ST21	ST21C2	*ehxA+/katP+/espP+/etpD-*	*stx1a*	-	beta1	NS	NS	+	+	-	281	283908	5507457	143	DRR103489	BEMW01000001-BEMW01000281
M06	10020	1996	human	Japan	ST21	ST21C2	*ehxA+/katP+/espP+/etpD-*	*stx1a*	-	beta1	NS	NS	+	+	-	301	285041	5693799	96	DRR103490	BEMX01000001-BEMX01000301
M07	10034	1997	human	Japan	ST21	ST21C2	*ehxA+/katP+/espP+/etpD-*	*stx1a*	-	beta1	NS	NS	+	+	-	308	230899	5632749	143	DRR103491	BEMY01000001-BEMY01000308
M08	10036	1997	human	Japan	ST21	ST21C2	*ehxA+/katP+/espP+/etpD-*	*stx1a*	-	beta1	NS	NS	-	-	-	332	354919	5678487	148	DRR103492	BEMZ01000001-BEMZ01000332
M09	10051	1997	human	Japan	ST21	ST21C1	*ehxA+/katP+/espP+/etpD-*	*stx1a*	-	beta1	NS	NS	+	-	-	387	244443	5667164	166	DRR103493	BENA01000001-BENA01000387
M10	10057	1997	human	Japan	ST21	ST21C2	*ehxA+/katP+/espP+/etpD-*	*stx1a*	-	beta1	NS	NS	+	+	-	316	354677	5550786	156	DRR103494	BENB01000001-BENB01000316
M11	10060	1998	human	Japan	ST21	ST21C2	*ehxA+/katP+/espP+/etpD-*	*stx1a*	-	beta1	NS	NS	+	-	-	343	230839	5513189	179	DRR103495	BENC01000001-BENC01000343
M12	10062	1998	human	Japan	ST21	ST21C2	*ehxA+/katP+/espP+/etpD-*	*stx1a*	-	beta1	NS	NS	+	+	-	301	256577	5412871	173	DRR103496	BEND01000001-BEND01000301
M13	10097	1998	human	Japan	ST21	ST21C2	*ehxA+/katP+/espP+/etpD-*	*stx1a*	-	beta1	NS	NS	+	-	-	282	282484	5576220	131	DRR103497	BENE01000001-BENE01000282
M14	10098	1998	human	Japan	ST21	ST21C2	*ehxA+/katP+/espP+/etpD-*	*stx1a*	-	beta1	NS	NS	+	+	-	324	358046	5395830	162	DRR103498	BENF01000001-BENF01000324
M15	10099	1998	human	Japan	ST21	ST21C2	*ehxA+/katP+/espP+/etpD-*	*stx1a*	-	beta1	NS	NS	+	-	-	332	267939	5382632	193	DRR103499	BENG01000001-BENG01000332
M16	10129	1999	human	Japan	ST21	ST21C2	*ehxA+/katP+/espP+/etpD-*	*stx1a*	-	beta1	NS	NS	+	+	-	348	244462	5626916	115	DRR103500	BENH01000001-BENH01000348
M17	10138	1999	human	Japan	ST21	ST21C2	*ehxA+/katP+/espP+/etpD-*	*stx1a*	-	beta1	NS	NS	+	+	-	366	230027	5706364	102	DRR103501	BENI01000001-BENI01000366
M18	10150	1999	human	Japan	ST21	ST21C2	*ehxA+/katP+/espP+/etpD-*	*stx1a*	-	beta1	NS	NS	+	+	-	306	284029	5598005	153	DRR103502	BENJ01000001-BENJ01000306
M19	10153	1999	human	Japan	ST21	ST21C2	*ehxA+/katP+/espP+/etpD-*	*stx1a*	*stx2a*	beta1	NS	NS	+	+	-	352	206509	5738667	125	DRR103503	BENK01000001-BENK01000352
M20	10169	1999	human	Japan	ST21	ST21C2	*ehxA+/katP+/espP+/etpD-*	*stx1a*	-	beta1	NS	NS	+	+	-	309	355015	5449669	129	DRR103504	BENL01000001-BENL01000309
M21	10174	1999	human	Japan	ST21	ST21C2	*ehxA+/katP+/espP+/etpD-*	*stx1a*	-	beta1	NS	NS	+	+	-	321	230642	5628714	124	DRR103505	BENM01000001-BENM01000321
M22	10182	1999	human	Japan	ST21	ST21C1	*ehxA+/katP+/espP+/etpD-*	*stx1a*	-	beta1	NS	NS	+	+	-	401	270347	5714186	113	DRR103506	BENN01000001-BENN01000401
M23	10184	1999	human	Japan	ST21	ST21C2	*ehxA+/katP+/espP+/etpD-*	*stx1a*	-	beta1	NS	NS	+	+	-	283	231148	5509775	141	DRR103507	BENO01000001-BENO01000283
M24	10201	2000	human	Japan	ST21	ST21C2	*ehxA+/katP+/espP+/etpD-*	*stx1a*	-	beta1	NS	NS	+	-	-	364	244227	5626215	148	DRR103508	BENP01000001-BENP01000364
M25	10207	2000	human	Japan	ST21	ST21C2	*ehxA+/katP+/espP+/etpD-*	*stx1a*	-	beta1	NS	NS	+	-	-	328	355252	5551005	159	DRR103509	BENQ01000001-BENQ01000328
M26	10236	2001	human	Japan	ST21	ST21C2	*ehxA+/katP+/espP+/etpD-*	*stx1a*	-	beta1	NS	NS	+	-	-	333	230666	5646509	149	DRR103510	BENR01000001-BENR01000333
M27	10239	2001	human	Japan	ST21	ST21C2	*ehxA+/katP+/espP+/etpD-*	*stx1a*	-	beta1	NS	NS	+	+	-	298	273315	5560961	172	DRR103511	BENS01000001-BENS01000298
M29	10246	2001	human	Japan	ST21	ST21C2	*ehxA+/katP+/espP+/etpD-*	*stx1a*	-	beta1	NS	NS	+	+	-	312	250561	5458627	123	DRR103512	BENT01000001-BENT01000312
M30	10257	2001	human	Japan	ST21	ST21C2	*ehxA+/katP+/espP+/etpD-*	*stx1a*	-	beta1	NS	NS	+	+	-	285	230718	5527880	152	DRR103513	BENU01000001-BENU01000285

M31	10308	2003	human	Japan	ST21	ST21C1	*ehxA+/katP+/espP+/etpD-*	*stx1a*	-	beta1	NS	NS	+	+	-	334	244491	5513922	142	DRR103514	BENV01000001-BENV01000334
M32	10312	2003	human	Japan	ST21	ST21C2	*ehxA-/katP+/espP-/etpD-*	*stx1a*	-	beta1	NS	NS	+	+	-	301	250483	5557394	145	DRR103515	BENW01000001-BENW01000301
M33	10314	2003	human	Japan	ST21	ST21C2	*ehxA+/katP+/espP+/etpD-*	*stx1a*	*stx2a*	beta1	NS	NS	+	-	-	327	244513	5575403	136	DRR103516	BENX01000001-BENX01000327
M34	10323	2003	human	Japan	ST21	ST21C1	*ehxA+/katP+/espP+/etpD-*	*stx1a*	-	beta1	NS	NS	+	+	-	355	244530	5577066	157	DRR103517	BENY01000001-BENY01000355
M35	10327	2003	human	Japan	ST21	ST21C1	*ehxA+/katP+/espP+/etpD-*	*stx1a*	-	beta1	NS	NS	-	-	-	436	230704	5777622	114	DRR103518	BENZ01000001-BENZ01000436
M36	10334	2003	human	Japan	ST21	ST21C2	*ehxA+/katP+/espP+/etpD-*	*stx1a*	-	beta1	NS	NS	+	-	-	410	230876	5719512	178	DRR103519	BEOA01000001-BEOA01000410
M37	10337	2003	human	Japan	ST21	ST21C1	*ehxA+/katP+/espP+/etpD-*	*stx1a*	-	beta1	NS	NS	NS	NS	NS	375	229237	5552729	128	DRR103520	BEOB01000001-BEOB01000375
M38	10338	2003	human	Japan	ST21	ST21C2	*ehxA+/katP+/espP+/etpD-*	*stx1a*	-	beta1	NS	NS	+	-	-	359	261688	5682920	140	DRR103521	BEOC01000001-BEOC01000359
M39	10367	2004	human	Japan	ST21	ST21C2	*ehxA+/katP+/espP+/etpD-*	*stx1a*	-	beta1	NS	NS	+	+	-	317	230666	5572546	144	DRR103522	BEOD01000001-BEOD01000317
M40	10368	2004	human	Japan	ST21	ST21C2	*ehxA+/katP+/espP+/etpD-*	*stx1a*	-	beta1	NS	NS	+	+	-	282	291105	5585860	114	DRR103523	BEOE01000001-BEOE01000282
M41	10369	2004	human	Japan	ST21	ST21C2	*ehxA+/katP+/espP+/etpD-*	*stx1a*	-	beta1	NS	NS	+	-	-	320	291209	5431033	123	DRR103524	BEOF01000001-BEOF01000320
M42	10373	2004	human	Japan	ST21	ST21C1	*ehxA+/katP+/espP+/etpD-*	*stx1a*	-	beta1	NS	NS	+	+	-	383	230639	5654913	110	DRR103525	BEOG01000001-BEOG01000383
M43	10374	2004	human	Japan	ST21	ST21C1	*ehxA+/katP+/espP+/etpD-*	*stx1a*	-	beta1	NS	NS	+	-	-	390	244514	5800455	86	DRR103526	BEOH01000001-BEOH01000390
M44	10379	2004	human	Japan	ST21	ST21C2	*ehxA+/katP+/espP+/etpD-*	*stx1a*	-	beta1	NS	NS	-	-	-	309	230488	5578204	121	DRR103527	BEOI01000001-BEOI01000309
M45	10401	2005	human	Japan	ST21	ST21C2	*ehxA+/katP+/espP+/etpD-*	*stx1a*	-	beta1	NS	NS	+	+	-	327	273267	5590551	132	DRR103528	BEOJ01000001-BEOJ01000327
M46	10405	2005	human	Japan	ST21	ST21C2	*ehxA+/katP+/espP+/etpD-*	*stx1a*	-	beta1	NS	NS	+	-	-	404	355039	5636839	135	DRR103529	BEOK01000001-BEOK01000404
M47	10419	2005	human	Japan	ST21	ST21C2	*ehxA+/katP+/espP+/etpD-*	*stx1a*	-	beta1	NS	NS	+	-	-	320	354885	5520251	120	DRR103530	BEOL01000001-BEOL01000320
M48	10421	2005	human	Japan	ST21	ST21C2	*ehxA+/katP+/espP+/etpD-*	*stx1a*	-	beta1	NS	NS	+	-	-	303	355093	5525854	126	DRR103531	BEOM01000001-BEOM01000303
M53	10426	2005	human	Japan	ST21	ST21C2	*ehxA+/katP+/espP+/etpD-*	*stx1a*	-	beta1	NS	NS	+	-	-	338	354312	5459397	117	DRR103532	BEON01000001-BEON01000338
M55	10430	2005	human	Japan	ST21	ST21C2	*ehxA+/katP+/espP+/etpD-*	*stx1a*	-	beta1	NS	NS	+	-	-	347	250433	5550222	148	DRR103533	BEOO01000001-BEOO01000347
M56	10437	2005	human	Japan	ST21	ST21C2	*ehxA+/katP+/espP+/etpD-*	*stx1a*	-	beta1	NS	NS	+	-	-	340	355101	5517001	120	DRR103534	BEOP01000001-BEOP01000340
M58	10442	2005	human	Japan	ST21	ST21C2	*ehxA+/katP+/espP+/etpD-*	*stx1a*	-	beta1	NS	NS	+	-	-	268	230728	5500674	118	DRR103535	BEOQ01000001-BEOQ01000268
M59	10443	2005	human	Japan	ST21	ST21C1	*ehxA+/katP+/espP+/etpD-*	*stx1a*	-	beta1	NS	NS	+	-	-	378	228431	5610778	154	DRR103536	BEOR01000001-BEOR01000378
M60	10444	2005	human	Japan	ST21	ST21C2	*ehxA+/katP+/espP+/etpD-*	*stx1a*	-	beta1	NS	NS	+	-	-	327	230525	5652494	169	DRR103537	BEOS01000001-BEOS01000327
M64	10473	2005	human	Japan	ST21	ST21C2	*ehxA+/katP+/espP+/etpD-*	*stx1a*	-	beta1	NS	NS	+	-	-	299	204666	5670253	119	DRR103538	BEOT01000001-BEOT01000299
M65	10480	2006	human	Japan	ST21	ST21C2	*ehxA+/katP+/espP+/etpD-*	*stx1a*	-	beta1	NS	NS	+	-	-	435	228886	5761178	148	DRR103539	BEOU01000001-BEOU01000435
M66	10481	2006	human	Japan	ST21	ST21C2	*ehxA+/katP+/espP+/etpD-*	*stx1a*	-	beta1	NS	NS	+	+	-	425	237935	5828969	197	DRR103540	BEOV01000001-BEOV01000425
M67	10488	2006	human	Japan	ST21	ST21C2	*ehxA+/katP+/espP+/etpD-*	*stx1a*	-	beta1	NS	NS	+	+	-	355	256570	5540455	165	DRR103541	BEOW01000001-BEOW01000355
M71	10524	2006	human	Japan	ST21	ST21C2	*ehxA+/katP+/espP+/etpD-*	*stx1a*	-	beta1	NS	NS	+	-	-	331	254194	5546337	171	DRR103542	BEOX01000001-BEOX01000331
M72	10536	2006	human	Japan	ST21	ST21C2	*ehxA+/katP+/espP+/etpD-*	*stx1a*	-	beta1	NS	NS	+	+	-	304	250478	5506565	145	DRR103543	BEOY01000001-BEOY01000304
M73	10542	2006	human	Japan	ST21	ST21C2	*ehxA+/katP+/espP+/etpD-*	*stx1a*	-	beta1	NS	NS	+	-	-	353	256572	5482574	175	DRR103544	BEOZ01000001-BEOZ01000353
M75	10572	2006	human	Japan	ST21	ST21C1	*ehxA+/katP+/espP+/etpD-*	*stx1a*	-	beta1	NS	NS	+	-	-	367	266450	5696583	114	DRR103545	BEPA01000001-BEPA01000367
M76	10574	2006	human	Japan	ST21	ST21C2	*ehxA+/katP+/espP+/etpD-*	*stx1a*	-	beta1	NS	NS	+	-	-	302	354913	5480896	134	DRR103546	BEPB01000001-BEPB01000302
M78	10591	2006	human	Japan	ST21	ST21C2	*ehxA+/katP+/espP+/etpD-*	*stx1a*	-	beta1	NS	NS	+	+	-	313	230484	5622264	111	DRR103547	BEPC01000001-BEPC01000313
M79	10594	2007	human	Japan	ST21	ST21C2	*ehxA+/katP+/espP+/etpD-*	*stx1a*	-	beta1	NS	NS	+	-	-	347	230927	5579963	126	DRR103548	BEPD01000001-BEPD01000347
M80	10650	2007	human	Japan	ST21	ST21C1	*ehxA+/katP+/espP+/etpD-*	*stx1a*	-	beta1	NS	NS	+	-	-	361	244355	5613596	145	DRR103549	BEPE01000001-BEPE01000361
M81	10795	2009	human	Japan	ST21	ST21C1	*ehxA+/katP+/espP+/etpD-*	*stx1a*	-	beta1	NS	NS	+	+	-	407	229400	5906059	107	DRR103550	BEPF01000001-BEPF01000407
M82	10808	2009	human	Japan	ST21	ST21C2	*ehxA+/katP+/espP+/etpD-*	*stx1a*	-	beta1	NS	NS	+	-	-	328	230829	5613546	127	DRR103551	BEPG01000001-BEPG01000328
M86	10811	2009	human	Japan	ST21	ST21C2	*ehxA+/katP+/espP+/etpD-*	*stx1a*	-	beta1	NS	NS	+	-	-	319	354980	5522393	116	DRR103552	BEPH01000001-BEPH01000319
M87	10815	2009	human	Japan	ST21	ST21C2	*ehxA+/katP+/espP+/etpD-*	*stx1a*	-	beta1	NS	NS	-	-	-	288	354495	5675848	123	DRR103553	BEPI01000001-BEPI01000288
M88	10865	2010	human	Japan	ST21	ST21C2	*ehxA+/katP+/espP+/etpD-*	*stx1a*	-	beta1	NS	NS	+	+	-	318	254682	5496923	110	DRR103554	BEPJ01000001-BEPJ01000318
M89	10872	2010	human	Japan	ST21	ST21C2	*ehxA+/katP+/espP+/etpD-*	*stx1a*	-	beta1	NS	NS	-	-	-	353	250784	5464636	102	DRR103555	BEPK01000001-BEPK01000353
M90	10873	2010	human	Japan	ST21	ST21C2	*ehxA+/katP+/espP+/etpD-*	*stx1a*	-	beta1	NS	NS	-	-	-	298	299884	5405839	122	DRR103556	BEPL01000001-BEPL01000298
M91	10875	2010	human	Japan	ST21	ST21C1	*ehxA+/katP+/espP+/etpD-*	*stx1a*	-	beta1	NS	NS	+	-	-	346	251220	5652031	149	DRR103557	BEPM01000001-BEPM01000346
M92	10892	2010	human	Japan	ST21	ST21C1	*ehxA+/katP+/espP+/etpD-*	*stx1a*	-	beta1	NS	NS	+	+	-	311	251055	5649694	117	DRR103558	BEPN01000001-BEPN01000311
M93	10924	2011	human	Japan	ST21	ST21C2	*ehxA+/katP+/espP+/etpD-*	*stx1a*	-	beta1	NS	NS	+	-	-	337	355111	5529550	140	DRR103559	BEPO01000001-BEPO01000337
M94	10938	2011	human	Japan	ST21	ST21C2	*ehxA+/katP+/espP+/etpD-*	*stx1a*	-	beta1	NS	NS	+	-	-	315	324322	5657363	108	DRR103560	BEPP01000001-BEPP01000315
M95	10948	2011	human	Japan	ST21	ST21C1	*ehxA+/katP+/espP+/etpD-*	*stx1a*	-	beta1	NS	NS	+	-	-	408	286300	5672105	102	DRR103561	BEPQ01000001-BEPQ01000408
O01	PV44	1996	human	Japan	ST21	ST21C2	*ehxA+/katP+/espP+/etpD-*	*stx1a*	-	beta1	11	F	+	+	-	291	250304	5333738	122	DRR103562	BEPR01000001-BEPR01000291
O02	PV230	1997	human	Japan	ST21	ST21C2	*ehxA+/katP+/espP+/etpD-*	*stx1a*	-	beta1	8	M	+	+	-	320	354611	5486759	108	DRR103563	BEPS01000001-BEPS01000320
O03	PV243	1997	human	Japan	ST21	ST21C2	*ehxA+/katP+/espP+/etpD-*	*stx1a*	-	beta1	5	F	+	-	-	303	354685	5427820	113	DRR103564	BEPT01000001-BEPT01000303
O04	PV253	1997	human	Japan	ST21	ST21C2	*ehxA+/katP+/espP+/etpD-*	*stx1a*	-	beta1	3	F	+	+	-	297	256429	5533149	81	DRR103565	BEPU01000001-BEPU01000297
O05	PV302	1997	human	Japan	ST21	ST21C1	*ehxA+/katP+/espP+/etpD-*	*stx1a*	-	beta1	1	M	+	-	-	306	230369	5643715	134	DRR103566	BEPV01000001-BEPV01000306
O06	PV306	1997	human	Japan	ST21	ST21C1	*ehxA-/katP-/espP-/etpD-*	-	-	beta1	5	F	+	-	-	260	244586	5305557	128	DRR103567	BEPW01000001-BEPW01000260
O07	PV428	1997	human	Japan	ST21	ST21C2	*ehxA+/katP-/espP-/etpD-*	*stx1a*	-	beta1	1	M	+	-	-	260	283232	5422259	115	DRR103568	BEPX01000001-BEPX01000260
O08	PV484	1998	human	Japan	ST21	ST21C2	*ehxA+/katP+/espP+/etpD-*	*stx1a*	-	beta1	6	F	+	-	-	300	230788	5526742	99	DRR103569	BEPY01000001-BEPY01000300
O09	PV520	1998	human	Japan	ST21	ST21C1	*ehxA+/katP+/espP+/etpD-*	-	-	beta1	2	M	+	+	-	195	345967	5307912	112	DRR103570	BEPZ01000001-BEPZ01000195
O10	PV542	1998	human	Japan	ST21	ST21C2	*ehxA-/katP-/espP-/etpD-*	*stx1a*	-	beta1	4	M	+	-	-	252	250369	5295182	106	DRR103571	BEQA01000001-BEQA01000252
O11	PV662	1998	human	Japan	ST21	ST21C2	*ehxA+/katP+/espP+/etpD-*	*stx1a*	-	beta1	2	F	+	-	-	257	230346	5421107	101	DRR103572	BEQB01000001-BEQB01000257
O12	PV679	1998	human	Japan	ST21	ST21C2	*ehxA-/katP-/espP-/etpD-*	*stx1a*	-	beta1	3	M	+	-	-	285	244091	5536238	93	DRR103573	BEQC01000001-BEQC01000285
O13	PV728	1998	human	Japan	ST21	ST21C2	*ehxA+/katP+/espP+/etpD-*	*stx1a*	-	beta1	17	F	+	-	-	270	243651	5490033	106	DRR103574	BEQD01000001-BEQD01000270
O16	PV773	1999	human	Japan	ST21	ST21C1	*ehxA+/katP+/espP+/etpD-*	*stx1a*	-	beta1	11	M	+	+	-	274	249862	5567078	83	DRR103575	BEQE01000001-BEQE01000274
O17	PV821	1999	human	Japan	ST21	ST21C2	*ehxA+/katP+/espP+/etpD-*	*stx1a*	-	beta1	7	M	+	+	-	284	354465	5448105	100	DRR103576	BEQF01000001-BEQF01000284
O18	PV835	1999	human	Japan	ST21	ST21C2	*ehxA+/katP+/espP+/etpD-*	*stx1a*	-	beta1	25	F	+	-	-	253	256825	5441876	125	DRR103577	BEQG01000001-BEQG01000253
O19	PV888	1999	human	Japan	ST21	ST21C2	*ehxA+/katP+/espP+/etpD-*	*stx1a*	-	beta1	1	M	+	+	-	278	286360	5553381	142	DRR103578	BEQH01000001-BEQH01000278
O20	PV942	1999	human	Japan	ST21	ST21C2	*ehxA+/katP+/espP+/etpD-*	*stx1a*	-	beta1	4	M	+	+	-	275	267561	5438979	113	DRR103579	BEQI01000001-BEQI01000275
O21	PV0010	2000	human	Japan	ST21	ST21C2	*ehxA+/katP+/espP+/etpD-*	*stx1a*	-	beta1	4	M	+	-	-	289	270064	5398847	110	DRR103580	BEQJ01000001-BEQJ01000289

O22	PV0029	2000	human	Japan	ST21	ST21C2	*ehxA+/katP+/espP+/etpD-*	*stx1a*	-	beta1	2	F	+	-	-	284	249858	5502321	100	DRR103581	BEQK01000001-BEQK01000284
O23	PV0041	2000	human	Japan	ST21	ST21C2	*ehxA+/katP+/espP+/etpD-*	*stx1a*	-	beta1	18	F	+	+	-	302	354749	5509826	91	DRR103582	BEQL01000001-BEQL01000302
O24	PV0042	2000	human	Japan	ST21	ST21C2	*ehxA+/katP+/espP+/etpD-*	*stx1a*	-	beta1	1	F	+	+	-	299	256562	5506015	88	DRR103583	BEQM01000001-BEQM01000299
O25	PV00132	2000	human	Japan	ST21	ST21C2	*ehxA+/katP+/espP+/etpD-*	*stx1a*	-	beta1	12	F	+	+	-	267	256807	5490200	103	DRR103584	BEQN01000001-BEQN01000267
O26	PV00166	2000	human	Japan	ST21	ST21C2	*ehxA+/katP+/espP+/etpD-*	*stx1a*	-	beta1	4	M	+	-	-	284	277085	5645838	108	DRR103585	BEQO01000001-BEQO01000284
O28	PV00176	2000	human	Japan	ST21	ST21C2	*ehxA+/katP+/espP+/etpD-*	*stx1a*	-	beta1	3	M	+	+	-	255	297387	5422569	144	DRR103586	BEQP01000001-BEQP01000255
O29	PV00199	2000	human	Japan	ST21	ST21C2	*ehxA+/katP+/espP+/etpD-*	*stx1a*	-	beta1	22	F	+	+	-	321	297431	5557523	97	DRR103587	BEQQ01000001-BEQQ01000321
O30	PV0174	2001	human	Japan	ST21	ST21C2	*ehxA+/katP+/espP+/etpD-*	*stx1a*	-	beta1	12	F	+	-	-	283	355063	5426101	100	DRR103588	BEQR01000001-BEQR01000283
O31	PV0182	2001	human	Japan	ST21	ST21C2	*ehxA+/katP+/espP+/etpD-*	*stx1a*	-	beta1	2	F	+	+	-	277	249848	5429124	95	DRR103589	BEQS01000001-BEQS01000277
O32	PV0198	2001	human	Japan	ST21	ST21C1	*ehxA+/katP+/espP+/etpD-*	*stx1a*	-	beta1	1	M	+	-	-	306	266156	5699477	81	DRR103590	BEQT01000001-BEQT01000306
O33	PV01131	2001	human	Japan	ST21	ST21C2	*ehxA+/katP+/espP+/etpD-*	*stx1a*	-	beta1	10	M	+	-	-	273	230657	5519048	112	DRR103591	BEQU01000001-BEQU01000273
O34	PV01169	2001	human	Japan	ST21	ST21C2	*ehxA+/katP+/espP+/etpD-*	*stx1a*	-	beta1	14	M	+	-	-	257	270995	5411491	118	DRR103592	BEQV01000001-BEQV01000257
O35	PV01198	2001	human	Japan	ST21	ST21C1	*ehxA+/katP+/espP+/etpD-*	-	-	beta1	5	M	+	-	-	277	249012	5411814	93	DRR103593	BEQW01000001-BEQW01000277
O36	PV01203	2001	human	Japan	ST21	ST21C2	*ehxA+/katP+/espP+/etpD-*	*stx1a*	-	beta1	1	F	+	+	-	286	354611	5482497	109	DRR103594	BEQX01000001-BEQX01000286
O37	PV01207	2001	human	Japan	ST21	ST21C2	*ehxA+/katP+/espP+/etpD-*	*stx1a*	-	beta1	1	M	+	+	-	299	282120	5468158	100	DRR103595	BEQY01000001-BEQY01000299
O38	PV01271	2001	human	Japan	ST21	ST21C2	*ehxA+/katP+/espP+/etpD-*	*stx1a*	-	beta1	2	F	+	-	-	319	252954	5546328	108	DRR103596	BEQZ01000001-BEQZ01000319
O39	PV029	2002	human	Japan	ST21	ST21C1	*ehxA+/katP+/espP+/etpD-*	*stx1a*	-	beta1	7	F	+	+	-	280	244536	5605756	110	DRR103597	BERA01000001-BERA01000280
O40	PV02100	2002	human	Japan	ST21	ST21C2	*ehxA+/katP+/espP+/etpD-*	*stx1a*	-	beta1	2	F	+	+	-	284	249875	5410877	87	DRR103598	BERB01000001-BERB01000284
O41	PV02105	2002	human	Japan	ST21	ST21C1	*ehxA+/katP+/espP+/etpD-*	*stx1a*	*stx2a*	beta1	3	M	+	+	-	254	244494	5599882	137	DRR103599	BERC01000001-BERC01000254
O42	PV02120	2002	human	Japan	ST21	ST21C2	*ehxA+/katP+/espP+/etpD-*	*stx1a*	-	beta1	4	M	+	+	-	310	250280	5493781	123	DRR103600	BERD01000001-BERD01000310
O43	PV02136	2002	human	Japan	ST21	ST21C2	*ehxA+/katP+/espP+/etpD-*	*stx1a*	-	beta1	3	M	+	+	-	281	298561	5522278	122	DRR103601	BERE01000001-BERE01000281
O44	PV033	2003	human	Japan	ST21	ST21C2	*ehxA-/katP-/espP-/etpD-*	*stx1a*	-	beta1	6	M	+	-	-	322	230537	5575884	129	DRR103602	BERF01000001-BERF01000322
O45	PV0382	2003	human	Japan	ST21	ST21C2	*ehxA-/katP-/espP-/etpD-*	*stx1a*	-	beta1	2	M	+	+	-	294	297411	5376296	146	DRR103603	BERG01000001-BERG01000294
O46	PV03100	2003	human	Japan	ST21	ST21C2	*ehxA+/katP+/espP+/etpD-*	*stx1a*	-	beta1	8	M	+	-	-	299	277100	5529531	144	DRR103604	BERH01000001-BERH01000299
O47	PV04129	2004	human	Japan	ST21	ST21C2	*ehxA+/katP+/espP+/etpD-*	*stx1a*	-	beta1	10	M	+	+	-	265	324756	5617178	123	DRR103605	BERI01000001-BERI01000265
O48	PV0519	2005	human	Japan	ST21	ST21C1	*ehxA+/katP+/espP+/etpD-*	*stx1a*	-	beta1	14	M	+	-	-	309	231272	5569361	135	DRR103606	BERJ01000001-BERJ01000309
O49	PV0664	2006	human	Japan	ST21	ST21C2	*ehxA+/katP+/espP+/etpD-*	*stx1a*	-	beta1	2	F	+	+	-	276	230770	5471397	154	DRR103607	BERK01000001-BERK01000276
O50	PV06134	2006	human	Japan	ST21	ST21C2	*ehxA+/katP+/espP+/etpD-*	*stx1a*	-	beta1	2	F	+	+	-	312	249987	5742196	112	DRR103608	BERL01000001-BERL01000312
O51	PV0715	2007	human	Japan	ST21	ST21C2	*ehxA+/katP+/espP+/etpD-*	*stx1a*	-	beta1	12	M	+	-	-	277	256570	5443231	163	DRR103609	BERM01000001-BERM01000277
O53	PV0817	2008	human	Japan	ST21	ST21C2	*ehxA+/katP+/espP+/etpD-*	*stx1a*	-	beta1	13	F	+	-	-	324	230302	5607220	118	DRR103610	BERN01000001-BERN01000324
O54	PV0813	2008	human	Japan	ST21	ST21C2	*ehxA+/katP+/espP+/etpD-*	*stx1a*	-	beta1	21	M	+	-	-	285	256478	5659503	110	DRR103611	BERO01000001-BERO01000285
O55	PV0818	2008	human	Japan	ST21	ST21C1	*ehxA+/katP+/espP+/etpD-*	*stx1a*	-	beta1	22	M	+	-	-	297	253700	5523101	132	DRR103612	BERP01000001-BERP01000297
O56	PV0838	2008	human	Japan	ST21	ST21C2	*ehxA+/katP+/espP+/etpD-*	*stx1a*	*stx2d*	beta1	12	F	+	-	-	313	250313	5536704	112	DRR103613	BERQ01000001-BERQ01000313
O57	PV0954	2009	human	Japan	ST21	ST21C2	*ehxA+/katP+/espP+/etpD-*	*stx1a*	-	beta1	3	F	+	-	-	250	256748	5395637	143	DRR103614	BERR01000001-BERR01000250
O58	PV0967	2009	human	Japan	ST21	ST21C1	*ehxA+/katP+/espP+/etpD-*	*stx1a*	-	beta1	36	M	+	-	-	267	347940	5545624	108	DRR103615	BERS01000001-BERS01000267
O59	PV09116	2009	human	Japan	ST21	ST21C2	*ehxA+/katP-/espP+/etpD-*	*stx1a*	-	beta1	4	M	+	-	-	298	242997	5481875	109	DRR103616	BERT01000001-BERT01000298
O60	PV105	2010	human	Japan	ST21	ST21C1	*ehxA-/katP-/espP-/etpD-*	*stx1a*	-	beta1	NS	NS	-	-	-	342	266410	5983989	116	DRR103617	BERU01000001-BERU01000342
O61	PV10116	2010	human	Japan	ST21	ST21C2	*ehxA+/katP+/espP+/etpD-*	*stx1a*	-	beta1	2	M	+	-	-	328	250469	5459585	109	DRR103618	BERV01000001-BERV01000328
O62	PV116	2011	human	Japan	ST21	ST21C2	*ehxA+/katP+/espP+/etpD-*	*stx1a*	-	beta1	5	F	+	-	-	304	256403	5464136	107	DRR103619	BERW01000001-BERW01000304
O63	PV119	2011	human	Japan	ST21	ST21C2	*ehxA+/katP+/espP+/etpD-*	*stx1a*	-	beta1	40	F	+	-	-	307	256549	5717377	120	DRR103620	BERX01000001-BERX01000307
O64	PV1122	2011	human	Japan	ST21	ST21C1	*ehxA+/katP+/espP+/etpD-*	*stx1a*	-	beta1	4	M	+	+	-	312	256344	5725110	103	DRR103621	BERY01000001-BERY01000312
O65	PV1125	2011	human	Japan	ST21	ST21C2	*ehxA+/katP+/espP+/etpD-*	*stx1a*	-	beta1	5	F	+	-	-	376	354913	5873541	82	DRR103622	BERZ01000001-BERZ01000376
O66	PV1176	2011	human	Japan	ST21	ST21C2	*ehxA-/katP-/espP-/etpD-*	*stx1a*	-	beta1	35	M	-	-	-	353	233983	5613098	94	DRR103623	BESA01000001-BESA01000353
O67	PV1179	2011	human	Japan	ST21	ST21C1	*ehxA+/katP+/espP+/etpD-*	*stx1a*	-	beta1	41	M	-	-	-	364	285463	5620665	98	DRR103624	BESB01000001-BESB01000364
O68	PV1196	2011	human	Japan	ST21	ST21C2	*ehxA+/katP+/espP+/etpD-*	*stx1a*	-	beta1	15	F	+	-	-	355	354829	5570430	140	DRR103625	BESC01000001-BESC01000355
O69	PV1195	2011	human	Japan	ST21	ST21C1	*ehxA+/katP+/espP+/etpD-*	*stx1a*	-	beta1	1	M	+	-	-	387	230911	5802190	133	DRR103626	BESD01000001-BESD01000387
O70	PV1191	2011	human	Japan	ST21	ST21C2	*ehxA+/katP+/espP+/etpD-*	*stx1a*	-	beta1	65	M	-	-	-	337	354902	5613258	95	DRR103627	BESE01000001-BESE01000337
P01	EH2035	2011	human	Belgium	ST21	ST21C1	*ehxA-/katP-/espP+/etpD-*	*stx1a*	-	beta1	72	M	+	-	-	414	293061	5914979	128	DRR103628	BESF01000001-BESF01000414
P02	EH2042	2011	human	Belgium	ST21	ST21C2	*ehxA+/katP+/espP+/etpD-*	*stx1a*	-	beta1	85	F	+	+	-	290	256694	5573567	88	DRR103629	BESG01000001-BESG01000290
P03	EH2057	2011	human	Belgium	ST21	ST21C1	*ehxA+/katP+/espP+/etpD-*	*stx1a*	-	beta1	2	M	+	+	-	292	250354	5495218	82	DRR103630	BESH01000001-BESH01000292
P04	EH2068	2011	human	Belgium	ST21	ST21C1	*ehxA+/katP+/espP+/etpD-*	*stx1a*	-	beta1	0	M	+	+	-	327	257078	5649375	102	DRR103631	BESI01000001-BESI01000327
P05	EH2075	2011	human	Belgium	ST21	ST21C1	*ehxA+/katP+/espP+/etpD-*	*stx1a*	-	beta1	3	M	+	+	-	346	243884	5536661	91	DRR103632	BESJ01000001-BESJ01000346
P06	EH2083	2011	human	Belgium	ST21	ST21C1	*ehxA+/katP+/espP+/etpD-*	*stx1a*	-	beta1	34	F	+	+	-	272	256686	5728173	79	DRR103633	BESK01000001-BESK01000272
P07	EH2115	2011	human	Belgium	ST21	ST21C1	*ehxA+/katP+/espP+/etpD-*	*stx1a*	-	beta1	1	M	+	-	-	333	230700	5650958	105	DRR103634	BESL01000001-BESL01000333
P08	EH2135	2012	human	Belgium	ST21	ST21C1	*ehxA+/katP+/espP+/etpD-*	*stx1a*	-	beta1	76	M	+	+	-	385	249926	5821545	90	DRR103635	BESM01000001-BESM01000385
P09	EH2149	2012	human	Belgium	ST21	ST21C1	*ehxA+/katP+/espP+/etpD-*	-	*stx2a*	beta1	1	F	+	-	+	299	254434	5490140	104	DRR103636	BESN01000001-BESN01000299
P10	EH2200	2012	human	Belgium	ST21	ST21C1	*ehxA+/katP+/espP+/etpD-*	*stx1a*	-	beta1	51	M	+	+	-	356	249769	5857958	78	DRR103637	BESO01000001-BESO01000356
P11	EH2208	2012	human	Belgium	ST21	ST21C1	*ehxA+/katP+/espP+/etpD-*	*stx1a*	-	beta1	8	M	+	-	-	335	231332	5703722	126	DRR103638	BESP01000001-BESP01000335
P12	EH2209	2012	human	Belgium	ST1705	ST21C1	*ehxA+/katP+/espP+/etpD-*	*stx1a*	-	beta1	3	F	+	-	-	300	250146	5658643	130	DRR103639	BESQ01000001-BESQ01000300
P13	EH2219	2012	human	Belgium	ST21	ST21C1	*ehxA+/katP+/espP+/etpD-*	*stx1a*	-	beta1	0	M	+	-	-	338	230701	5724051	80	DRR103640	BESR01000001-BESR01000338
P14	EH2244	2012	human	Belgium	ST21	ST21C1	*ehxA+/katP+/espP+/etpD-*	*stx1a*	-	beta1	0	F	+	-	-	321	256695	5548774	51	DRR103641	BESS01000001-BESS01000321
P15	EH2251	2013	human	Belgium	ST21	ST21C1	*ehxA+/katP+/espP+/etpD-*	*stx1a*	-	beta1	21	F	+	+	-	298	297703	5553525	97	DRR103642	BEST01000001-BEST01000298
P16	EH2252	2013	human	Belgium	ST21	ST21C1	*ehxA+/katP+/espP+/etpD-*	*stx1a*	-	beta1	4	F	+	+	-	335	199627	5797260	41	DRR103643	BESU01000001-BESU01000335
P17	EH2257	2013	human	Belgium	new	ST21C1	*ehxA+/katP+/espP+/etpD-*	*stx1a*	-	beta1	68	F	+	+	-	300	244402	5649202	45	DRR103644	BESV01000001-BESV01000300
P18	EH2258	2013	human	Belgium	ST1705	ST21C1	*ehxA+/katP+/espP+/etpD-*	*stx1a*	-	beta1	82	M	+	-	-	259	250135	5520259	55	DRR103645	BESW01000001-BESW01000259
She01	KS22-2	2013	bovine	Japan	ST21	ST21C2	*ehxA+/katP+/espP+/etpD-*	*stx1a*	-	beta1	NS	NS	NS	NS	NS	239	244493	5505669	30	DRR103646	BESX01000001-BESX01000239
She02	KS32	2013	bovine	Japan	new	ST29C3	*ehxA-/katP-/espP-/etpD-*	-	-	beta1	NS	NS	NS	NS	NS	170	259015	5549566	45	DRR103647	BESY01000001-BESY01000170

She03	KS78	2013	bovine	Japan	ST21	ST21C1	*ehxA+/katP+/espP+/etpD-*	-	-	beta1	NS	NS	NS	NS	NS	240	286671	5664833	43	DRR103648	BESZ01000001-BESZ01000240
She04	KS180	2013	bovine	Japan	ST21	ST21C1	*ehxA+/katP+/espP+/etpD-*	-	-	beta1	NS	NS	NS	NS	NS	271	238495	5757177	37	DRR103649	BETA01000001-BETA01000271
She05	KS181	2013	bovine	Japan	ST21	ST21C1	*ehxA+/katP+/espP+/etpD-*	-	-	beta1	NS	NS	NS	NS	NS	252	230073	5766939	42	DRR103650	BETB01000001-BETB01000252
She06	KS266	2013	bovine	Japan	ST21	ST21C2	*ehxA+/katP+/espP+/etpD-*	*stx1a*	-	beta1	NS	NS	NS	NS	NS	266	244431	5530346	29	DRR103651	BETC01000001-BETC01000266
She07	KS327	2013	bovine	Japan	ST21	ST21C1	*ehxA+/katP+/espP+/etpD-*	-	-	beta1	NS	NS	NS	NS	NS	236	238734	5796289	41	DRR103652	BETD01000001-BETD01000236
She08	KS334	2013	bovine	Japan	ST21	ST21C1	*ehxA+/katP+/espP+/etpD-*	-	-	beta1	NS	NS	NS	NS	NS	231	366998	5789172	57	DRR103653	BETE01000001-BETE01000231
T01	1219	1996	human	Japan	ST21	ST21C2	*ehxA+/katP+/espP+/etpD-*	-	-	beta1	NS	NA	NA	NA	NA	313	232759	5425513	135	DRR103654	BETF01000001-BETF01000313
T02	1291	1996	human	Japan	ST21	ST21C2	*ehxA+/katP+/espP+/etpD-*	*stx1a*	-	beta1	2	male	+	+	-	349	354465	5475654	102	DRR103655	BETG01000001-BETG01000349
T03	1388	1996	human	Japan	ST21	ST21C2	*ehxA+/katP+/espP+/etpD-*	*stx1a*	-	beta1	NS	female	NA	NA	NA	309	229930	5467908	113	DRR103656	BETH01000001-BETH01000309
T04	1447	1997	human	Japan	ST21	ST21C2	*ehxA+/katP+/espP+/etpD-*	*stx1a*	-	beta1	3	male	+	-	-	330	230886	5533043	117	DRR103657	BETI01000001-BETI01000330
T05	1449	1997	human	Japan	ST21	ST21C2	*ehxA+/katP+/espP+/etpD-*	*stx1a*	-	beta1	3	male	+	-	-	311	285886	5432972	118	DRR103658	BETJ01000001-BETJ01000311
T06	1460	1997	human	Japan	ST21	ST21C2	*ehxA-/katP-/espP-/etpD-*	*stx1a*	-	beta1	1	male	+	-	-	283	256779	5356970	107	DRR103659	BETK01000001-BETK01000283
T08	1485	1997	human	Japan	ST21	ST21C2	*ehxA+/katP+/espP+/etpD-*	*stx1a*	-	beta1	1	male	+	-	-	325	244381	5496753	112	DRR103660	BETL01000001-BETL01000325
T09	1520	1998	human	Japan	ST21	ST21C2	*ehxA-/katP-/espP-/etpD-*	*stx1a*	-	beta1	5	male	+	+	-	369	265151	5562667	111	DRR103661	BETM01000001-BETM01000369
T11	1602	1998	human	Japan	ST21	ST21C1	*ehxA+/katP+/espP+/etpD-*	*stx1a*	*stx2a*	beta1	3	female	+	+	+	232	244586	5463572	117	DRR103662	BETN01000001-BETN01000232
T12	1603	1998	human	Japan	ST21	ST21C2	*ehxA+/katP+/espP+/etpD-*	*stx1a*	-	beta1	8	female	+	+	-	376	282430	5589646	69	DRR103663	BETO01000001-BETO01000376
T13	1639	1998	human	Japan	ST21	ST21C2	*ehxA+/katP+/espP+/etpD-*	*stx1a*	-	beta1	NS	male	+	-	-	331	250404	5518630	142	DRR103664	BETP01000001-BETP01000331
T14	1640	1998	human	Japan	ST21	ST21C2	*ehxA+/katP+/espP+/etpD-*	*stx1a*	-	beta1	4	male	+	+	-	327	230433	5541861	97	DRR103665	BETQ01000001-BETQ01000327
T15	1657	1998	human	Japan	ST29	ST29C3	*ehxA-/katP-/espP-/etpD-*	-	-	beta1	NS	female	NA	NA	NA	231	258327	5511760	114	DRR103666	BETR01000001-BETR01000231
T16	1671	1998	human	Japan	ST21	ST21C2	*ehxA+/katP+/espP+/etpD-*	*stx1a*	-	beta1	49	female	-	-	-	317	246494	5605962	85	DRR103667	BETS01000001-BETS01000317
T17	1674	1998	human	Japan	ST21	ST21C2	*ehxA-/katP-/espP-/etpD-*	*stx1a*	-	beta1	NS	male	NA	NA	NA	301	303751	5486809	106	DRR103668	BETT01000001-BETT01000301
T18	1677	1998	human	Japan	ST21	ST21C2	*ehxA+/katP+/espP+/etpD-*	*stx1a*	-	beta1	NS	male	+	+	-	339	297617	5443034	113	DRR103669	BETU01000001-BETU01000339
T19	1678	1998	human	Japan	ST21	ST21C2	*ehxA+/katP+/espP+/etpD-*	*stx1a*	-	beta1	2	male	+	-	-	319	250416	5524903	124	DRR103670	BETV01000001-BETV01000319
T20	1715	1999	human	Japan	ST21	ST21C2	*ehxA+/katP+/espP+/etpD-*	*stx1a*	-	beta1	6	male	+	-	-	384	267415	5606157	93	DRR103671	BETW01000001-BETW01000384
T21	1720	1999	human	Japan	ST21	ST21C2	*ehxA+/katP+/espP+/etpD-*	*stx1a*	-	beta1	13	female	+	-	-	383	324313	5593201	87	DRR103672	BETX01000001-BETX01000383
T22	1750	1999	human	Japan	ST21	ST21C2	*ehxA+/katP+/espP+/etpD-*	*stx1a*	-	beta1	5	male	+	-	-	356	273294	5517235	75	DRR103673	BETY01000001-BETY01000356
T23	1771	1999	human	Japan	ST21	ST21C2	*ehxA+/katP+/espP+/etpD-*	-	-	beta1	4	male	+	-	-	292	250492	5403451	144	DRR103674	BETZ01000001-BETZ01000292
T24	1794	2000	human	Japan	ST21	ST21C2	*ehxA-/katP-/espP-/etpD-*	*stx1a*	-	beta1	89	male	+	-	-	415	230383	5519362	115	DRR103675	BEUA01000001-BEUA01000415
T25	1798	2000	human	Japan	ST21	ST21C2	*ehxA-/katP-/espP-/etpD-*	*stx1a*	-	beta1	32	female	-	-	-	382	250418	5473623	110	DRR103676	BEUB01000001-BEUB01000382
T26	1822	2000	human	Japan	ST21	ST21C2	*ehxA+/katP+/espP+/etpD-*	*stx1a*	-	beta1	3	male	+	-	-	346	229820	5461797	76	DRR103677	BEUC01000001-BEUC01000346
T27	1950	2001	human	Japan	ST21	ST21C2	*ehxA+/katP+/espP+/etpD-*	*stx1a*	-	beta1	57	male	-	-	-	328	250369	5454570	111	DRR103678	BEUD01000001-BEUD01000328
T29	1958	2001	human	Japan	ST21	ST21C1	*ehxA+/katP+/espP+/etpD-*	*stx1a*	*stx2a*	beta1	2	male	+	+	-	362	230806	5567582	150	DRR103679	BEUE01000001-BEUE01000362
T30	2038	2001	human	Japan	ST21	ST21C1	*ehxA+/katP+/espP+/etpD-*	*stx1a*	-	beta1	11	male	+	+	-	361	231340	5683655	160	DRR103680	BEUF01000001-BEUF01000361
T31	2046	2001	human	Japan	ST21	ST21C2	*ehxA+/katP+/espP+/etpD-*	*stx1a*	-	beta1	3	female	+	-	-	296	244472	5471141	134	DRR103681	BEUG01000001-BEUG01000296
T32	2054	2001	human	Japan	ST21	ST21C2	*ehxA+/katP+/espP+/etpD-*	*stx1a*	*stx2a*	beta1	10	female	+	-	-	353	314278	5505794	102	DRR103682	BEUH01000001-BEUH01000353
T33	2153	2002	human	Japan	ST21	ST21C2	*ehxA+/katP+/espP+/etpD-*	*stx1a*	-	beta1	1	male	+	-	-	345	277074	5750535	140	DRR103683	BEUI01000001-BEUI01000345
T34	2154	2002	human	Japan	ST21	ST21C2	*ehxA+/katP+/espP+/etpD-*	*stx1a*	-	beta1	30	male	+	-	-	306	250063	5388510	143	DRR103684	BEUJ01000001-BEUJ01000306
T35	2156	2002	human	Japan	ST21	ST21C2	*ehxA+/katP+/espP+/etpD-*	*stx1a*	-	beta1	10	female	+	-	-	360	354892	5536619	157	DRR103685	BEUK01000001-BEUK01000360
T37	2159	2002	human	Japan	ST21	ST21C2	*ehxA+/katP+/espP+/etpD-*	*stx1a*	-	beta1	26	male	+	-	-	363	256841	5548331	110	DRR103686	BEUL01000001-BEUL01000363
T39	2191	2002	human	Japan	ST21	ST21C2	*ehxA+/katP+/espP+/etpD-*	*stx1a*	-	beta1	3	female	+	+	-	336	231059	5571622	124	DRR103687	BEUM01000001-BEUM01000336
T40	2204	2003	human	Japan	ST21	ST21C2	*ehxA+/katP+/espP+/etpD-*	*stx1a*	-	beta1	23	female	+	-	-	363	256457	5440154	112	DRR103688	BEUN01000001-BEUN01000363
T41	2243	2003	human	Japan	ST21	ST21C2	*ehxA+/katP+/espP+/etpD-*	*stx1a*	-	beta1	3	female	+	+	-	290	249981	5445975	79	DRR103689	BEUO01000001-BEUO01000290
T42	2313	2004	human	Japan	ST21	ST21C1	*ehxA+/katP+/espP+/etpD-*	*stx1a*	-	beta1	40	female	-	-	-	374	244514	5559359	70	DRR103690	BEUP01000001-BEUP01000374
T43	2314	2004	human	Japan	ST21	ST21C1	*ehxA+/katP+/espP+/etpD-*	*stx1a*	-	beta1	16	female	+	+	-	331	250986	5517909	129	DRR103691	BEUQ01000001-BEUQ01000331
T44	2318	2004	human	Japan	ST21	ST21C2	*ehxA+/katP-/espP+/etpD-*	*stx1a*	-	beta1	18	male	+	+	-	307	250431	5545853	83	DRR103692	BEUR01000001-BEUR01000307
T45	2329	2004	human	Japan	ST21	ST21C2	*ehxA-/katP-/espP-/etpD-*	*stx1a*	-	beta1	8	male	+	-	-	280	247320	5482206	98	DRR103693	BEUS01000001-BEUS01000280
T46	2342	2004	human	Japan	ST21	ST21C2	*ehxA+/katP+/espP+/etpD-*	*stx1a*	-	beta1	49	female	-	-	-	297	256777	5441353	94	DRR103694	BEUT01000001-BEUT01000297
T47	2418	2005	human	Japan	ST21	ST21C2	*ehxA+/katP+/espP+/etpD-*	*stx1a*	-	beta1	5	male	+	-	-	314	277074	5601571	105	DRR103695	BEUU01000001-BEUU01000314
T48	2442	2005	human	Japan	ST21	ST21C2	*ehxA+/katP+/espP+/etpD-*	*stx1a*	-	beta1	29	male	+	-	-	365	245371	5653630	102	DRR103696	BEUV01000001-BEUV01000365
T49	2591	2006	human	Japan	ST21	ST21C2	*ehxA+/katP+/espP+/etpD-*	*stx1a*	-	beta1	5	female	+	+	-	294	250331	5609315	117	DRR103697	BEUW01000001-BEUW01000294
T50	2592	2006	human	Japan	ST21	ST21C2	*ehxA+/katP+/espP+/etpD-*	*stx1a*	-	beta1	57	female	+	+	-	311	354726	5493904	92	DRR103698	BEUX01000001-BEUX01000311
T51	2594	2006	human	Japan	ST21	ST21C1	*ehxA+/katP+/espP+/etpD-*	*stx1a*	-	beta1	44	female	-	-	-	359	230547	5676096	111	DRR103699	BEUY01000001-BEUY01000359
T52	2597	2006	human	Japan	ST21	ST21C2	*ehxA+/katP+/espP+/etpD-*	*stx1a*	-	beta1	20	male	+	+	-	336	247311	5534118	124	DRR103700	BEUZ01000001-BEUZ01000336
T53	2598	2006	human	Japan	ST21	ST21C2	*ehxA+/katP+/espP+/etpD-*	*stx1a*	*stx2a*	beta1	98	female	+	-	-	317	291071	5499176	150	DRR103701	BEVA01000001-BEVA01000317
T57	2694	2006	human	Japan	ST21	ST21C2	*ehxA+/katP+/espP+/etpD-*	*stx1a*	-	beta1	5	male	+	-	-	347	256612	5576692	110	DRR103702	BEVB01000001-BEVB01000347
T58	2706	2006	human	Japan	ST21	ST21C2	*ehxA+/katP+/espP+/etpD-*	*stx1a*	-	beta1	58	female	+	+	-	292	250691	5495773	118	DRR103703	BEVC01000001-BEVC01000292
T59	2833	2007	human	Japan	ST21	ST21C2	*ehxA+/katP+/espP+/etpD-*	*stx1a*	-	beta1	50	female	-	-	-	269	256872	5437159	140	DRR103704	BEVD01000001-BEVD01000269
T60	2840	2007	human	Japan	ST21	ST21C2	*ehxA+/katP+/espP+/etpD-*	*stx1a*	-	beta1	6	female	+	-	-	302	357578	5514974	142	DRR103705	BEVE01000001-BEVE01000302
T61	2847	2007	human	Japan	ST21	ST21C2	*ehxA+/katP+/espP+/etpD-*	*stx1a*	-	beta1	1	male	+	-	-	395	247017	5533678	148	DRR103706	BEVF01000001-BEVF01000395
T62	2932	2008	human	Japan	ST21	ST21C2	*ehxA+/katP+/espP+/etpD-*	*stx1a*	-	beta1	52	male	+	-	-	286	269518	5314463	147	DRR103707	BEVG01000001-BEVG01000286
T63	2935	2008	human	Japan	ST21	ST21C2	*ehxA+/katP+/espP+/etpD-*	*stx1a*	-	beta1	2	male	+	-	-	402	354912	5516732	185	DRR103708	BEVH01000001-BEVH01000402
T64	2944	2008	human	Japan	ST21	ST21C2	*ehxA+/katP+/espP+/etpD-*	*stx1a*	-	beta1	3	female	+	+	-	335	256524	5466783	154	DRR103709	BEVI01000001-BEVI01000335
T65	2966	2008	human	Japan	ST21	ST21C2	*ehxA+/katP+/espP+/etpD-*	*stx1a*	-	beta1	14	female	+	+	-	325	235031	5519659	167	DRR103710	BEVJ01000001-BEVJ01000325
T66	3034	2009	human	Japan	ST21	ST21C2	*ehxA+/katP+/espP+/etpD-*	*stx1a*	-	beta1	8	female	+	+	-	298	256727	5552452	103	DRR103711	BEVK01000001-BEVK01000298
T67	3052	2009	human	Japan	ST21	ST21C2	*ehxA+/katP+/espP+/etpD-*	*stx1a*	-	beta1	11	male	+	+	-	298	244238	5551521	116	DRR103712	BEVL01000001-BEVL01000298
T68	3186	2011	human	Japan	ST21	ST21C2	*ehxA+/katP+/espP+/etpD-*	*stx1a*	-	beta1	20	female	-	-	-	351	230832	5622642	89	DRR103713	BEVM01000001-BEVM01000351
T69	3240	2011	human	Japan	ST21	ST21C1	*ehxA+/katP+/espP+/etpD-*	*stx1a*	-	beta1	63	female	+	-	-	317	266351	5603487	156	DRR103714	BEVN01000001-BEVN01000317

T70	3244	2011	human	Japan	ST21	ST21C2	*ehxA+/katP+/espP+/etpD-*	*stx1a*	-	beta1	72	male	+	-	-	336	230480	5503660	141	DRR103715	BEVO01000001-BEVO01000336
T71	3260	2011	human	Japan	ST21	ST21C2	*ehxA+/katP+/espP+/etpD-*	*stx1a*	-	beta1	24	male	-	-	-	299	267430	5513770	141	DRR103716	BEVP01000001-BEVP01000299
T72	3277	2011	human	Japan	ST21	ST21C2	*ehxA+/katP+/espP+/etpD-*	*stx1a*	-	beta1	2	male	+	-	-	350	249934	5457434	127	DRR103717	BEVQ01000001-BEVQ01000350
T73	3297	2011	human	Japan	ST21	ST21C2	*ehxA+/katP+/espP+/etpD-*	*stx1a*	-	beta1	62	female	-	-	-	324	256394	5449821	116	DRR103718	BEVR01000001-BEVR01000324
YB01	18-150	2006	bovine	Japan	ST21	ST21C2	*ehxA+/katP+/espP+/etpD-*	*stx1a*	-	beta1	NS	NS	NS	NS	NS	283	256718	5574193	45	DRR103719	BEVS01000001-BEVS01000283
YB02	18-145	2006	bovine	Japan	ST21	ST21C2	*ehxA+/katP+/espP+/etpD-*	*stx1a*	-	beta1	NS	NS	NS	NS	NS	248	256716	5434494	63	DRR103720	BEVT01000001-BEVT01000248
YB03	20-45-4	2008	bovine	Japan	ST21	ST21C1	*ehxA+/katP+/espP+/etpD-*	*stx1a*	-	beta1	NS	NS	NS	NS	NS	340	237602	5990187	52	DRR103721	BEVU01000001-BEVU01000340
YB05	21-67-1	2009	bovine	Japan	ST21	ST21C2	*ehxA+/katP+/espP+/etpD-*	*stx1a*	-	beta1	NS	NS	NS	NS	NS	273	256463	5612673	65	DRR103722	BEVV01000001-BEVV01000273
YB06	21-67-5	2009	bovine	Japan	ST21	ST21C1	*ehxA+/katP+/espP+/etpD-*	*stx1a*	-	beta1	NS	NS	NS	NS	NS	272	297466	5749075	62	DRR103723	BEVW01000001-BEVW01000272
YB13	21-85-13	2009	bovine	Japan	ST21	ST21C2	*ehxA+/katP+/espP+/etpD-*	*stx1a*	-	beta1	NS	NS	NS	NS	NS	304	354970	5734936	66	DRR103724	BEVX01000001-BEVX01000304

NS: not specified/Unkown

Table S2. O26 genome sequence data obtained from public database

Strain	ST	lineage	*plasmid gene profile*	*stx1*	*stx2*	intimin (eaeA) subtype	Sorce	Yr isolated	Country	accession No. (WGS)	accession No. (SRA)
4131	ST29	ST29C3	*ehxA-/katP-/espP-/etpD-*	-	-	beta1	cattle	2011	USA	LPYO00000000	
4170	ST29	ST29C3	*ehxA-/katP-/espP-/etpD-*	-	-	beta1	cattle	2011	USA	LPYP00000000	
4196	ST29	ST29C3	*ehxA-/katP-/espP-/etpD-*	-	-	beta1	cattle	2011	USA	LPYQ00000000	
4513	ST29	ST29C3	*ehxA-/katP-/espP-/etpD-*	-	-	beta1	cattle	2011	USA	LPYY00000000	
11368	ST21	ST21C2	*ehxA+/katP+/espP+/etpD-*	*stx1a*	-	beta1	human	2001	Japan	NC_013369	
21765	ST29	ST29C3	*ehxA-/katP-/espP-/etpD-*	-	*stx2a*	beta1	human	2005	France	CDLB00000000	
34827	ST29	ST29C3	*ehxA-/katP-/espP-/etpD-*	-	*stx2a*	beta1	human	2012	Francs	LDXF01000000	
34870	ST29	ST29C3	*ehxA-/katP-/espP-/etpD-*	-	*stx2a*	beta1	human	2012	Francs	LDXE01000000	
36084	ST21	ST21C1	*ehxA+/katP+/espP+/etpD-*	-	*stx2a*	beta1	human	2013	Francs	LDXI01000000	
36293	ST29	ST29C3	*ehxA-/katP-/espP-/etpD-*	-	*stx2d*	beta1	human	2013	Francs	LDXC00000000	
36348	ST29	ST29C3	*ehxA-/katP-/espP-/etpD-*	-	*stx2d*	beta1	human	2013	Francs	LDXD00000000	
36493	ST29	ST29C3	*ehxA-/katP-/espP-/etpD-*	-	*stx2d*	beta1	human	2013	Francs	LDXB00000000	
36708	ST29	ST29C2	*ehxA+/katP-/espP-/etpD+*	-	*stx2a*	beta1	human	2013	Francs	LDXG01000000	
070293	ST21	ST21C1	*ehxA+/katP+/espP+/etpD-*	-	*stx2a*	beta1	human	2007	Japan	PRJDB5136	
070765	ST21	ST21C1	*ehxA+/katP+/espP+/etpD-*	-	*stx2a*	beta1	human	2007	Japan	PRJDB5136	
072720	ST21	ST21C2	*ehxA+/katP+/espP+/etpD-*	-	*stx2a*	beta1	human	2007	Japan	PRJDB5136	
080884	ST21	ST21C2	*ehxA+/katP+/espP+/etpD-*	*stx1a*	*stx2a*	beta1	human	2008	Japan	PRJDB5136	
082383	ST21	ST21C1	*ehxA+/katP+/espP+/etpD-*	*stx1a*	*stx2a*	beta1	human	2008	Japan	PRJDB5136	
090405	ST29	ST29C2	*ehxA+/katP-/espP-/etpD+*	-	*stx2a*	beta1	human	2009	Japan	PRJDB5136	
090532	ST21	ST21C1	*ehxA-/katP-/espP-/etpD-*	*stx1a*	*stx2a*	beta1	human	2009	Japan	PRJDB5136	
93270	ST21	ST21C1	*ehxA-/katP+/espP+/etpD-*	-	*stx2d*	beta1	human	2014	UK		SRR3241998
93279	ST21	ST21C1	*ehxA+/katP+/espP+/etpD-*	*stx1a*	*stx2a*	beta1	human	2014	UK		SRR3241986
93280	ST21	ST21C1	*ehxA+/katP+/espP+/etpD-*	-	*stx2a*	beta1	human	2014	UK		SRR3241985
93285	ST21	ST21C1	*ehxA+/katP+/espP+/etpD-*	-	*stx2a*	beta1	human	2014	UK		SRR3241859
93288	ST21	ST21C1	*ehxA+/katP+/espP+/etpD-*	*stx1a*	*stx2a*	beta1	human	2014	UK		SRR3241849
93302	ST21	ST21C1	*ehxA+/katP+/espP+/etpD-*	*stx1a*	-	beta1	human	2014	UK		SRR3241997
93304	ST21	ST21C2	*ehxA+/katP+/espP+/etpD-*	*stx1a*	-	beta1	human	2014	UK		SRR3240981
93306	ST21	ST21C1	*ehxA+/katP+/espP+/etpD-*	*stx1a*	*stx2a*	beta1	human	2014	UK		SRR3241852
93309	ST21	ST21C1	*ehxA+/katP+/espP+/etpD-*	*stx1a*	*stx2a*	beta1	human	2014	UK		SRR3240962
93310	ST21	ST21C1	*ehxA+/katP+/espP+/etpD-*	*stx1a*	*stx2a*	beta1	human	2014	UK		SRR3240963
93314	ST21	ST21C1	*ehxA+/katP+/espP+/etpD-*	*stx1a*	-	beta1	human	2014	UK		SRR3241868
93320	ST21	ST21C1	*ehxA+/katP+/espP+/etpD-*	*stx1a*	-	beta1	human	2014	UK		SRR3241978
93322	ST21	ST21C2	*ehxA+/katP+/espP+/etpD-*	*stx1a*	-	beta1	human	2014	UK		SRR3241839
101272	ST21	ST21C1	*ehxA+/katP+/espP+/etpD-*	*stx1a*	*stx2a*	beta1	human	2014	UK		SRR3241855
101358	ST29	ST29C1	*ehxA+/katP-/espP+/etpD-*	-	*stx2a*	beta1	human	2010	Japan	PRJDB5136	
102085	ST21	ST21C1	*ehxA+/katP+/espP+/etpD-*	*stx1a*	*stx2a*	beta1	human	2010	Japan	PRJDB5136	
111609	ST21	ST21C2	*ehxA+/katP+/espP+/etpD-*	*stx1a*	*stx2a*	beta1	human	2011	Japan	PRJDB5136	
112183	ST21	ST21C1	*ehxA+/katP+/espP+/etpD-*	*stx1a*	*stx2a*	beta1	human	2011	Japan	PRJDB5136	
112664	ST21	ST21C1	*ehxA-/katP-/espP-/etpD-*	*stx1a*	*stx2a*	beta1	human	2011	Japan	PRJDB5136	
121825	ST21	ST21C2	*ehxA+/katP+/espP+/etpD-*	-	*stx2a*	beta1	human	2012	Japan	PRJDB5136	
121840	ST21	ST21C1	*ehxA+/katP+/espP+/etpD-*	*stx1a*	*stx2a*	beta1	human	2012	Japan	PRJDB5136	
122147	ST21	ST21C1	*ehxA+/katP+/espP+/etpD-*	-	*stx2a*	beta1	human	2012	Japan	PRJDB5136	
122657	ST21	ST21C2	*ehxA+/katP+/espP+/etpD-*	*stx1a*	*stx2a*	beta1	human	2012	Japan	PRJDB5136	
122711	ST29	ST29C2	*ehxA+/katP-/espP-/etpD+*	-	*stx2a*	beta1	human	2012	Japan	PRJDB5136	
129381	ST21	ST21C1	*ehxA+/katP+/espP+/etpD-*	*stx1a*	*stx2a*	beta1	human	2015	UK		SRR3578565
130549	ST29	ST29C1	*ehxA+/katP-/espP+/etpD-*	-	*stx2a*	beta1	human	2013	Japan	PRJDB5136	
130562	ST29	ST29C1	*ehxA+/katP-/espP+/etpD-*	-	*stx2a*	beta1	human	2013	Japan	PRJDB5136	
131037	ST29	ST29C1	*ehxA+/katP-/espP+/etpD-*	-	*stx2a*	beta1	human	2013	Japan	PRJDB5136	
131458	ST21	ST21C1	*ehxA+/katP+/espP+/etpD-*	-	*stx2a*	beta1	human	2013	Japan	PRJDB5136	
132265	ST21	ST21C2	*ehxA+/katP+/espP+/etpD-*	*stx1a*	*stx2a*	beta1	human	2013	Japan	PRJDB5136	
132777	ST29	ST29C2	*ehxA+/katP-/espP-/etpD+*	-	*stx2a*	beta1	human	2013	Japan	PRJDB5136	
133042	ST21	ST21C1	*ehxA+/katP+/espP+/etpD-*	*stx1a*	*stx2a*	beta1	human	2013	Japan	PRJDB5136	
140921	ST21	ST21C2	*ehxA+/katP+/espP+/etpD-*	-	*stx2a*	beta1	human	2014	Japan	PRJDB5136	
141423	ST29	ST29C1	*ehxA+/katP-/espP+/etpD-*	-	*stx2a*	beta1	human	2014	Japan	PRJDB5136	
141424	ST21	ST21C2	*ehxA+/katP+/espP+/etpD-*	-	*stx2a*	beta1	human	2014	Japan	PRJDB5136	
141425	ST29	ST29C1	*ehxA+/katP-/espP+/etpD-*	-	*stx2a*	beta1	human	2014	Japan	PRJDB5136	
163205	ST21	ST21C1	*ehxA+/katP+/espP+/etpD-*	*stx1a*	-	beta1	human	2015	UK		SRR3579385
166268	ST21	ST21C1	*ehxA+/katP+/espP+/etpD-*	*stx1a*	-	beta1	human	2015	UK		SRR3530808
168308	ST21	ST21C2	*ehxA+/katP+/espP+/etpD-*	*stx1a*	-	beta1	human	2015	UK		SRR3578157

168310	ST21	ST21C1	*ehxA+/katP+/espP+/etpD-*	*stx1a*	-	beta1	human	2015	UK		SRR3581325
173581	ST21	ST21C1	*ehxA+/katP+/espP+/etpD-*	*stx1a*	-	beta1	human	2015	UK		SRR3581385
177415	ST21	ST21C1	*ehxA+/katP+/espP+/etpD-*	*stx1a*	-	beta1	human	2015	UK		SRR3579394
178621	ST21	ST21C1	*ehxA+/katP+/espP+/etpD-*	*stx1a*	-	beta1	human	2015	UK		SRR3581504
178924	ST21	ST21C1	*ehxA+/katP+/espP+/etpD-*	*stx1a*	*stx2a*	beta1	human	2015	UK		SRR3578990
179577	ST29	ST29C2	*ehxA+/katP-/espP-/etpD+*	-	-	beta1	human	2015	UK		SRR3581359
179803	ST21	ST21C1	*ehxA+/katP+/espP+/etpD-*	*stx1a*	-	beta1	human	2015	UK		SRR3581421
179808	ST21	ST21C1	*ehxA+/katP+/espP+/etpD-*	-	-	beta1	human	2015	UK		SRR3578655
184389	ST21	ST21C1	*ehxA+/katP+/espP+/etpD-*	*stx1a*	-	beta1	human	2015	UK		SRR3578772
189231	ST21	ST21C1	*ehxA+/katP+/espP+/etpD-*	*stx1a*	-	beta1	human	2015	UK		SRR3578573
190756	ST21	ST21C1	*ehxA+/katP+/espP+/etpD-*	*stx1a*	-	beta1	human	2015	UK		SRR3578572
190757	ST21	ST21C1	*ehxA+/katP+/espP+/etpD-*	*stx1a*	-	beta1	human	2015	UK		SRR3579364
194189	ST21	ST21C1	*ehxA+/katP+/espP+/etpD-*	*stx1a*	-	beta1	human	2015	UK		SRR3578943
194236	ST29	ST29C2	*ehxA+/katP-/espP-/etpD+*	-	*stx2a*	beta1	human	2015	UK		SRR3578935
194237	ST21	ST21C1	*ehxA+/katP+/espP+/etpD-*	-	*stx2d*	beta1	human	2015	UK		SRR3578941
194241	ST21	ST21C1	*ehxA+/katP+/espP+/etpD-*	-	*stx2a*	beta1	human	2015	UK		SRR3579365
194242	ST29	ST29C2	*ehxA+/katP-/espP-/etpD+*	-	*stx2a*	beta1	human	2015	UK		SRR3578581
195529	ST29	ST29C2	*ehxA+/katP-/espP-/etpD+*	-	*stx2a*	beta1	human	2015	UK		SRR3578643
195530	ST384	ST21C1	*ehxA+/katP+/espP+/etpD-*	-	*stx2a*	beta1	human	2015	UK		SRR3578617
195545	ST21	ST21C1	*ehxA+/katP+/espP+/etpD-*	-	*stx2a*	beta1	human	2015	UK		SRR3578292
224098	ST21	ST21C1	*ehxA+/katP+/espP+/etpD-*	*stx1a*	-	beta1	human	2016	UK		SRR3226393
227795	ST21	ST21C1	*ehxA+/katP-/espP-/etpD+*	*stx1a*	-	beta1	human	2016	UK		SRR3574335
238378	ST21	ST21C1	*ehxA+/katP+/espP+/etpD-*	*stx1a*	-	beta1	human	2016	UK		SRR3579373
248542	ST29	ST29other	*ehxA+/katP+/espP+/etpD-*	-	-	beta1	human	2016	UK		SRR3578908
03-3500	ST21	ST21C2	*ehxA+/katP+/espP+/etpD-*	*stx1a*	*stx2a*	beta1	human	2001	USA	JHNT00000000	
05-3646	ST21	ST21C1	*ehxA+/katP+/espP+/etpD-*	*stx1a*	-	beta1	human	2005	USA	JHOE01000000	SRR3794431
06-3464	ST21	ST21C2	*ehxA+/katP+/espP+/etpD-*	*stx1a*	-	beta1	human	2006	USA	JHNO00000000	SRR3213940
1270-A	ST29	ST29C3	*ehxA-/katP-/espP-/etpD-*	-	-	beta1	cattle	2011	USA	LPXY00000000	
1341-A	ST29	ST29C1	*ehxA+/katP-/espP+/etpD-*	-	-	beta1	cattle	2011	USA	LPYB00000000	
1357-A	ST29	ST29C3	*ehxA-/katP-/espP-/etpD-*	-	-	beta1	cattle	2011	USA	LPTW00000000	
165_12	ST21	ST21C1	*ehxA+/katP+/espP+/etpD-*	*stx1a*	*stx2a*	beta1	human	2012	UK		SRR2035370
1668-A-A	ST29	ST29C1	*ehxA+/katP-/espP+/etpD-*	-	-	beta1	cattle	2011	USA	LPYG00000000	
1676-A	ST29	ST29C3	*ehxA-/katP-/espP-/etpD-*	-	-	beta1	cattle	2011	USA	LPYC00000000	
1692-A	ST29	ST29C3	*ehxA-/katP-/espP-/etpD-*	-	-	beta1	cattle	2011	USA	LPYD00000000	
1740-A-A	ST29	ST29C3	*ehxA-/katP-/espP-/etpD-*	-	-	beta1	cattle	2011	USA	LPYE00000000	
1802-A	ST29	ST29C3	*ehxA-/katP-/espP-/etpD-*	-	*stx2a*	beta1	cattle	2011	USA	LPZD00000000	SRR3018395
181_09	ST21	ST21C1	*ehxA+/katP+/espP+/etpD-*	*stx1a*	*stx2a*	beta1	human	2009	UK		SRR2035432
1958-A-B	ST29	ST29C3	*ehxA-/katP-/espP-/etpD-*	-	-	beta1	cattle	2011	USA	LPYI00000000	
2009C-3612	ST29	ST29C1	*ehxA+/katP-/espP+/etpD-*	-	*stx2a*	beta1	human	2009	USA	JHGZ00000000	
2009C-3689	ST29	ST29C1	*ehxA+/katP-/espP+/etpD-*	-	*stx2a*	beta1	human	2009	USA	JHGX00000000	
2009C-3996	ST21	ST21C2	*ehxA+/katP+/espP+/etpD-*	*stx1a*	-	beta1	human	2009	USA	JHGV00000000	
2009C-4747	ST21	ST21C2	*ehxA+/katP+/espP+/etpD-*	*stx1a*	-	beta1	human	2009	USA	JHGM00000000	
2009C-4760	ST21	ST21C2	*ehxA+/katP+/espP+/etpD-*	*stx1a*	-	beta1	human	2009	USA	JHGK00000000	
2009C-4826	ST21	ST21C2	*ehxA+/katP+/espP+/etpD-*	*stx1a*	-	beta1	human	2009	USA	JHGI00000000	
2010C-3051	ST21	ST21C2	*ehxA+/katP+/espP+/etpD-*	*stx1a*	-	beta1	human	2010	USA	JHGA00000000	
2010C-3472	ST21	ST21C2	*ehxA+/katP+/espP+/etpD-*	*stx1a*	-	beta1	human	2010	USA	JHFX00000000	
2010C-3871	ST21	ST21C1	*ehxA+/katP+/espP+/etpD-*	*stx1a*	*stx2a*	beta1	human	2010	USA	JHFJ00000000	
2010C-3902	ST21	ST21C2	*ehxA+/katP+/espP+/etpD-*	*stx1a*	*stx2a*	beta1	human	2010	USA	JHFH00000000	
2010C-4244	ST21	ST21C2	*ehxA+/katP+/espP+/etpD-*	*stx1a*	-	beta1	human	2010	USA	JHFD00000000	
2010C-4347	ST21	ST21C2	*ehxA+/katP+/espP+/etpD-*	*stx1a*	-	beta1	human	2010	USA	JHFB00000000	
2010C-4430	ST21	ST21C2	*ehxA+/katP+/espP+/etpD-*	*stx1a*	-	beta1	human	2010	USA	JHND00000000	
2010C-4788	ST21	ST21C2	*ehxA+/katP+/espP+/etpD-*	*stx1a*	-	beta1	human	2010	USA	JHMS00000000	
2010C-4819	ST21	ST21C2	*ehxA+/katP+/espP+/etpD-*	*stx1a*	*stx2a*	beta1	human	2010	USA	JHMP00000000	
2010C-4834	ST21	ST21C2	*ehxA+/katP+/espP+/etpD-*	*stx1a*	-	beta1	human	2010	USA	JHMN00000000	
2010C-5028	ST21	ST21C2	*ehxA+/katP+/espP+/etpD-*	*stx1a*	-	beta1	human	2010	USA	JHMI00000000	
2010EL-1699	ST21	ST21C2	*ehxA+/katP+/espP+/etpD-*	*stx1a*	-	beta1	human	2010	USA	JHMF00000000	
2011C-3270	ST21	ST21C2	*ehxA+/katP+/espP+/etpD-*	*stx1a*	-	beta1	human	2011	USA	JHLY00000000	
2011C-3274	ST21	ST21C2	*ehxA+/katP+/espP+/etpD-*	*stx1a*	-	beta1	human	2011	USA	JAST00000000	
2011C-3282	ST21	ST21C2	*ehxA+/katP+/espP+/etpD-*	*stx1a*	-	beta1	human	2011	USA	JHLX00000000	
2011C-3387	ST21	ST21C2	*ehxA-/katP+/espP-/etpD-*	*stx1a*	-	beta1	human	2011	USA	JHLV00000000	
2011C-3506	ST21	ST21C1	*ehxA+/katP+/espP+/etpD-*	*stx1a*	-	beta1	human	2011	USA	JHLS00000000	
2011C-3655	ST21	ST21C2	*ehxA+/katP+/espP+/etpD-*	*stx1a*	-	beta1	human	2011	USA	JHLN00000000	

2105-G	ST29	ST29C3	*ehxA-/katP-/espP-/etpD-*	-	-	beta1	cattle	2011	USA	LPYK00000000	
2139-A	ST29	ST29C3	*ehxA-/katP-/espP-/etpD-*	-	-	beta1	cattle	2011	USA	LPYM00000000	
2152-B	ST29	ST29C3	*ehxA-/katP-/espP-/etpD-*	-	-	beta1	cattle	2011	USA	LPYA00000000	
2176-A	ST29	ST29C3	*ehxA-/katP-/espP-/etpD-*	-	-	beta1	cattle	2011	USA	LPYN00000000	
2194-B	ST29	ST29C3	*ehxA-/katP-/espP-/etpD-*	-	-	beta1	cattle	2011	USA	LPYJ00000000	
2223-B	ST29	ST29C3	*ehxA-/katP-/espP-/etpD-*	-	-	beta1	cattle	2011	USA	LPYL00000000	
2228-A	ST29	ST29C3	*ehxA-/katP-/espP-/etpD-*	-	-	beta1	cattle	2011	USA	LPXZ00000000	
2270-502_12	ST21	ST21C1	*ehxA+/katP+/espP+/etpD-*	*stx1a*	*stx2a*	beta1	human	2012	UK		SRR2035404
2290-502_12	ST29	ST29C3	*ehxA-/katP-/espP-/etpD-*	*stx1a*	-	beta1	human	2012	UK	SRR2035369	
3674-A	ST29	ST29C3	*ehxA-/katP-/espP-/etpD-*	-	-	beta1	cattle	2011	USA	LPZK00000000	
4271-C	ST29	ST29C3	*ehxA-/katP-/espP-/etpD-*	-	-	beta1	cattle	2011	USA	LPYU00000000	SRR3018384
4277-H-A	ST29	ST29C3	*ehxA-/katP-/espP-/etpD-*	-	-	beta1	cattle	2011	USA	LPYR00000000	
4368-A	ST29	ST29C3	*ehxA-/katP-/espP-/etpD-*	-	-	beta1	cattle	2011	USA	LPYS00000000	
4435-B	ST29	ST29C3	*ehxA-/katP-/espP-/etpD-*	-	-	beta1	cattle	2011	USA	LPYT00000000	
4468-A-A	ST29	ST29C3	*ehxA-/katP-/espP-/etpD-*	-	*stx2a*	beta1	cattle	2011	USA	LPZF00000000	
4592-A	ST29	ST29C3	*ehxA-/katP-/espP-/etpD-*	-	-	beta1	cattle	2011	USA	LPYZ00000000	
461_09	ST21	ST21C1	*ehxA+/katP+/espP+/etpD-*	*stx1a*	*stx2a*	beta1	human	2009	UK		SRR2036124
467_10	ST21	ST21C1	*ehxA+/katP+/espP+/etpD-*	-	*stx2a*	beta1	human	2010	UK		SRR2120768
4730-C	ST29	ST29C3	*ehxA-/katP-/espP-/etpD-*	-	-	beta1	cattle	2011	USA	LPYX00000000	
482_12	ST21	ST21C1	*ehxA+/katP+/espP+/etpD-*	-	*stx2a*	beta1	human	2012	UK		SRR2035364
4822-A	ST29	ST29C3	*ehxA-/katP-/espP-/etpD-*	-	*stx2a*	beta1	cattle	2011	USA	LPZE00000000	
4848-A-B	ST29	ST29C3	*ehxA-/katP-/espP-/etpD-*	-	-	beta1	cattle	2011	USA	LPYW00000000	
4860-A	ST29	ST29C3	*ehxA-/katP-/espP-/etpD-*	-	*stx2a*	beta1	cattle	2011	USA	LPZG00000000	
4863-A	ST29	ST29C1	*ehxA+/katP-/espP+/etpD-*	-	*stx2a*	beta1	cattle	2011	USA	LPZH00000000	
5019-A	ST29	ST29C3	*ehxA-/katP-/espP-/etpD-*	-	*stx2a*	beta1	cattle	2011	USA	LPZJ00000000	
519_11	ST21	ST21C1	*ehxA+/katP+/espP+/etpD-*	*stx1a*	*stx2a*	beta1	human	2011	UK		SRR2120771
5196-B	ST29	ST29C3	*ehxA-/katP-/espP-/etpD-*	-	*stx2a*	beta1	cattle	2011	USA	LPZI00000000	
5206-E-B	ST29	ST29C3	*ehxA-/katP-/espP-/etpD-*	-	-	beta1	cattle	2011	USA	LPZC00000000	
5583-H	ST29	ST29C3	*ehxA-/katP-/espP-/etpD-*	-	-	beta1	cattle	2011	USA	LPZA00000000	
5687-B	ST29	ST29C3	*ehxA-/katP-/espP-/etpD-*	-	-	beta1	cattle	2011	USA	LPZB00000000	
605_10	ST21	ST21C1	*ehxA+/katP+/espP+/etpD-*	*stx1a*	*stx2a*	beta1	human	2010	UK		SRR2120773
624_12	ST21	ST21C1	*ehxA+/katP+/espP+/etpD-*	-	*stx2a*	beta1	human	2012	UK		SRR2120774
626_12	ST21	ST21C1	*ehxA+/katP+/espP+/etpD-*	-	*stx2a*	beta1	human	2012	UK		SRR2035399
627_12	ST21	ST21C1	*ehxA+/katP+/espP+/etpD-*	-	*stx2a*	beta1	human	2012	UK		SRR2035368
637_13	ST21	ST21C1	*ehxA+/katP+/espP+/etpD-*	-	*stx2a*	beta1	human	2013	UK		SRR2035374
670_13	ST29	ST29C3	*ehxA-/katP-/espP-/etpD-*	-	-	beta1	human	2013	UK		SRR2035376
680_13	ST29	ST29other	*ehxA+/katP+/espP-/etpD-*	-	-	beta1	human	2013	UK		SRR2035402
97-3250	ST21	ST21C1	*ehxA+/katP+/espP+/etpD-*	*stx1a*	*stx2a*	beta1	human	1997	USA	NZ_JHEW00000000	
ATCC BAA-219	ST21	ST21C1	*ehxA+/katP+/espP+/etpD-*	*stx1a*	*stx2a*	beta1	unknown	2003	USA	AYOF00000000	
CFSAN001629	ST21	ST21C2	*ehxA-/katP-/espP-/etpD-*	*stx1a*	-	beta1	human	1997	USA	NZ_AMXO00000000	
CVM10021	ST21	ST21C1	*ehxA+/katP+/espP+/etpD-*	*stx1a*	-	beta1	cow	1995	USA	AKAZ00000000	
CVM10026	ST21	ST21C1	*ehxA+/katP-/espP-/etpD+*	*stx1a*	-	beta1	cow	1995	USA	AJVX00000000	
CVM10030	ST21	ST21C2	*ehxA+/katP-/espP+/etpD-*	*stx1a*	-	beta1	cow	1995	USA	AKBA00000000	
CVM9942	ST21	ST21C1	*ehxA+/katP-/espP+/etpD-*	*stx1a*	-	beta1	cow	1983	USA	AJVW00000000	
CVM9952	ST21	ST21C2	*ehxA-/katP-/espP-/etpD-*	*stx1a*	-	beta1	pig	1985	USA	AKBC00000000	
STEC1117	ST21	ST21C1	*ehxA+/katP+/espP+/etpD-*	*stx1a*	-	beta1	human	2013	Netherlands	NZ_LOFU00000000	
STEC1236	ST21	ST21C2	*ehxA+/katP+/espP+/etpD-*	*stx1a*	-	beta1	human	2013	Netherlands	NZ_LOGB00000000	
STEC1293	ST21	ST21C1	*ehxA+/katP+/espP+/etpD-*	*stx1a*	*stx2a*	beta1	human	2013	Netherlands	NZ_LOGF00000000	
STEC2110.3	ST21	ST21C2	*ehxA+/katP+/espP+/etpD-*	-	-	beta1	human	2013	Netherlands	NZ_LOJE00000000	
STEC2144	ST21	ST21C2	*ehxA+/katP+/espP+/etpD-*	*stx1a*	-	beta1	human	2013	Netherlands	NZ_LOGU00000000	
STEC2346	ST21	ST21C2	*ehxA+/katP+/espP+/etpD-*	*stx1a*	-	beta1	human	2013	Netherlands	NZ_LOHA00000000	
STEC2920	ST21	ST21C1	*ehxA+/katP-/espP+/etpD-*	*stx1a*	-	beta1	human	2013	Netherlands	NZ_LOIU00000000	
STEC380	ST21	ST21C1	*ehxA+/katP+/espP+/etpD-*	*stx1a*	-	beta1	human	2013	Netherlands	NZ_LOCV00000000	
STEC477	ST21	ST21C2	*ehxA+/katP+/espP+/etpD-*	*stx1a*	-	beta1	human	2013	Netherlands	NZ_LOCY00000000	
STEC487	ST21	ST21C1	*ehxA+/katP+/espP+/etpD-*	*stx1a*	-	beta1	human	2013	Netherlands	NZ_LODA00000000	
STEC563	ST21	ST21C1	*ehxA+/katP+/espP+/etpD-*	*stx1a*	*stx2a*	beta1	human	2013	Netherlands	NZ_LODD00000000	
STEC709	ST21	ST21C1	*ehxA+/katP+/espP+/etpD-*	*stx1a*	*stx2a*	beta1	human	2013	Netherlands	NZ_LOFM00000000	
STEC764	ST21	ST21C2	*ehxA+/katP-/espP+/etpD-*	*stx1a*	-	beta1	human	2013	Netherlands	NZ_LOFP00000000	
STEC931	ST29	ST29C2	*ehxA+/katP-/espP-/etpD+*	-	*stx2a*	beta1	human	2013	Netherlands	NZ_LOFS00000000	

Among the strains with public genome information, several strains in which core genome alignment was completely identical to that of at least one another strain were removed from this table and further analysis.

Table S3. The locus_tag numbers of the T3SS effectors and plasmid-encoded VFs used as references in Fig. 3B.

ECO26_0889	*nleH*
ECO26_0890	*cif*
ECO26_0895	*nleG1*
ECO26_0898	*espJ*
ECO26_1162	*tccP*
ECO26_1163	*espV*
ECO26_1525	*espO*
ECO26_1526	*espK*
ECO26_1636	*nleG2*
ECO26_1637	*nleG3*
ECO26_1638	*nleG4*
ECO26_1639	*espW*
ECO26_1640	*nleG5*
ECO26_1641	*espM*
ECO26_1806	*nleG6*
ECO26_1807/8	*nleA*
ECO26_1810	*nleH*
ECO26_1811	*nleF*
ECO26_1814	*espO*
ECO26_1816	*nleG7*
ECO26_1817	*espM*
ECO26_1820	*nleG8*
ECO26_1976	*nleG9*
ECO26_1977	*nleG10*
ECO26_1978	*nleG11*
ECO26_2220	*nleC*
ECO26_2609	*nleG12*
ECO26_2610	*ibe*
ECO26_3149	*nleG13*
ECO26_3150	*nleG14*
ECO26_3663	*espK*
ECO26_3664	*espN*
ECO26_3665	*espX*
ECO26_5248	*ibe*
ECO26_5250	*espG*
ECO26_5268	*espZ*
ECO26_5275	*espH*
ECO26_5277	*map*
ECO26_5278	*tir*
ECO26_5285	*espB*
ECO26_5289	*espF*
ECO26_5293	*nleE*
ECO26_5294	*nleB*
ECO26_5295	*espL*
ECs0850	*nleD*
ECO111_1634	*ospG*
ECO26p1_02	*ehxA*
ECO26p1_25	*efa-1*
ECO26p1_46	*katP*
ECO26p1_89	*ecf1*
pO157_003	*etpD*
pO157_079	*espP*

Table S4. Strains classfied into minor or undifined STs

strain	ST	description
B37	ST1705	single locus variant of ST21
B43	ST1705	single locus variant of ST21
P12	ST1705	single locus variant of ST21
P18	ST1705	single locus variant of ST21
195530	ST384	single locus variant of ST21
P17	undifined ST	single locus variant of ST21
She02	undifined ST	single locus variant of ST29

Table S5. Conservation of the T3SS effector and plasmid virulence genes on each genomic element

genomic element	No. of effector gene	No. of strain		
		all positive	partially negative	all negative
Prophage P02	4	428	1	0
Prophage P03	2	329	27	73
Prophage P05	2	382	15	32
Prophage P06	6	330	96	3
Prophage P08	8	387	38	4
Prophage P09	2	331	93	5
Prophage P11	1	379	0	50
Prophage P14	2	423	2	4
Prophage P17	2	429	0	0
Prophage P19	3	373	28	28
LEE & accessory	11	425	3	1
plasmid	6	361	36	32

Table S6. Summary of distribution of acquired antibiotic resistane genes in each lineage of the O26 strains

	% of strains positive to resistance gene(s) for each antibiotc category*									
	Agly	Tmt	Bla	Pne	MLS	Sul	Tet	Fom	Flq	Cl
ST21_Cluster1 (n=153)	49.7	15.7	34.0	11.8	6.5	47.7	35.3	1.3	2.0	1.3
ST21_Cluster2 (n=243)	30.5	2.1	10.3	1.2	0.4	28.0	23.9	0.0	0.0	0.0
ST29 (n=33)	30.3	3.0	12.1	18.2	0.0	27.3	33.3	0.0	0.0	0.0

*AGly: aminoglycoside, Tmt: Trimethoprim, Bla: Beta-lactam, Phe: Phenicol, MLS: Macrolide-lincosamide-streptogramin, Sul: Sulfonamide, Tet: Tetracycline, Fom: Fosfomycin, Flq: Fluoroquinolone, Cl: Colistin
